# Graph convolutional network for modeling corridor-based urban transformation: Revealing spatial coherence in land-use change in Erbil

**DOI:** 10.1371/journal.pone.0357090

**Published:** 2026-08-27

**Authors:** Mustafa Aziz Amen, Hourakhsh Ahmad Nia, Amen Mustafa Aziz

**Affiliations:** 1 Department of Architecture, The American University of Kurdistan, Duhok, Iraq; 2 Department of Architecture, Faculty of Engineering and Natural Sciences, Alanya University, Alanya, Antalya, Türkiye; 3 Department of Artificial Intelligence, College of Computer and Informatics, Salahaddin University, Erbil, Iraq; Northeast Forestry University, CHINA

## Abstract

Residential-to-commercial transformation often occurs selectively along particular urban corridors, yet the structural mechanisms underlying this spatial concentration remain insufficiently understood. This study examines whether the historical configuration of Erbil’s street network explains corridor-based land-use change between 2004 and 2024. A reconstructed 2004 street network comprising 279 nodes was analyzed using weighted degree, betweenness, closeness, PageRank, and straightness centrality. Logistic Regression and a two-layer Graph Convolutional Network (GCN) were trained using identical predictors and evaluated through a stratified 70:30 split, 30 repeated random splits, conventional classification metrics, and spatial-coherence indicators. In the main split, the GCN achieved higher accuracy, precision, and F1-score, whereas Logistic Regression obtained slightly higher recall and ROC–AUC. Repeated validation confirmed that Logistic Regression remained more sensitive to transformed nodes, while the GCN produced fewer connected components, lower fragmentation, and higher largest-connected-component ratios. Weighted straightness emerged as the strongest and most consistent predictor of commercial conversion. The findings demonstrate that historical street-network structure meaningfully shapes later corridor transformation and that model evaluation should extend beyond node-level accuracy to include spatial continuity. The proposed framework offers a transferable tool for anticipating commercial expansion and supporting proactive corridor planning decisions.

## 1. Introduction

Urban land-use transformation is a fundamental process through which cities respond to changing economic conditions, transportation systems, and patterns of human activity. One of its most common manifestations is the gradual conversion of residential streets into commercial corridors, a process that influences accessibility, land value, travel behavior, and urban form. However, commercial transformation rarely occurs uniformly across the urban fabric. Instead, commercial activities often concentrate along selected streets, while adjacent streets with similar physical and morphological characteristics remain predominantly residential. Understanding the mechanisms responsible for this selective corridor-based transformation is therefore important for urban planning, commercial development, and sustainable land-use management, particularly in rapidly growing cities [1–9].

Street-network structure has long been recognized as an important determinant of urban development because accessibility, connectivity, and movement potential influence the spatial distribution of commercial activities and land-use intensity [8,10–15]. Previous studies have demonstrated significant associations between street-network centrality and retail concentration, economic activity, and commercial development; however, most have examined these relationships at the city or district scale using contemporary or cross-sectional data. Consequently, limited attention has been given to whether the configuration of a historical street network can explain why residential-to-commercial transformation later develops along one particular corridor while neighboring streets remain predominantly residential. Conventional land-use models, particularly Logistic Regression, provide interpretable estimates of how individual predictors influence the probability of urban transformation [18 –20], but they do not explicitly learn from the adjacency, connectivity, and relational dependence embedded within street networks [21 –23]. Graph-based models can address this limitation by combining node-level attributes with network topology [24], yet their application to historically grounded, corridor-specific land-use transformation remains insufficiently investigated. Therefore, the research gap concerns three related issues: the limited use of historical networks to explain later corridor transformation, the lack of direct comparisons between conventional statistical and graph-based models using identical predictors, and the insufficient evaluation of whether predicted land-use changes reproduce the connected spatial structure of an observed commercial corridor.

To address these gaps, this study reconstructs the historical street network of the selected Erbil corridor as it existed in 2004 and examines whether its structural characteristics explain the residential-to-commercial transformation observed by 2024. Logistic Regression and a Graph Convolutional Network are compared using the same weighted street-network centrality measures, allowing the additional contribution of graph-based relational learning to be evaluated against an interpretable statistical benchmark. Model performance is assessed not only through conventional node-level classification metrics, including accuracy, precision, recall, F1-score, and ROC–AUC, but also through spatial-coherence indicators that measure connected components, fragmentation, and the largest connected component ratio. This combined historical, comparative, and spatial evaluation framework directly addresses the identified gaps by linking prior street-network structure to later corridor transformation and by assessing whether model predictions reproduce the connected spatial form of the observed commercial corridor.

The study contributes to the literature in three ways. First, it provides a historically grounded explanation of corridor-based commercial transformation using the reconstructed street network that existed before the observed land-use change. Second, it presents a direct comparison between Logistic Regression and Graph Convolutional Networks using identical predictor variables within the same analytical framework. Third, it extends conventional model evaluation by incorporating spatial coherence measures, demonstrating that reproducing the connected spatial structure of urban transformation is an important dimension of predictive performance.

## 2. Literature Review

### 2.1. Street-network structure and commercial land-use transformation

Residential-to-commercial transformation is closely associated with the spatial organization of street networks. Classical urban economic theories explain commercial concentration in terms of accessibility, land value, and locational advantage [1,2]. More recent configurational approaches extend this perspective by demonstrating that the position of streets within the network strongly influences pedestrian movement, visibility, and commercial attractiveness [3–7]. Consequently, commercial corridors are increasingly understood as products of both economic forces and street-network configuration rather than simply responses to land demand.

Numerous empirical studies have confirmed significant relationships between street-network centrality and commercial development. Degree, betweenness, closeness, PageRank, and straightness centrality have been associated with retail concentration, commercial facilities, employment distribution, and land-use intensity in a variety of urban contexts [8,10–14]. Collectively, these studies demonstrate that streets occupying strategically important positions within the urban network are more likely to experience commercial growth because they provide greater accessibility, movement opportunities, and spatial exposure.

Although these findings establish the importance of street-network structure, most existing studies examine commercial development at the city or district scale. Consequently, they explain general spatial trends rather than the selective transformation of individual corridors. Much less attention has been devoted to understanding why one corridor experiences commercial conversion while neighboring streets with similar physical characteristics remain predominantly residential. This corridor-scale perspective represents an important gap in the current literature.

### 2.2. Logistic regression

Within the broader land-use change literature, logistic regression remains one of the most widely used methods for modeling binary urban transformation [15,16]. Its appeal lies in its interpretability, enabling researchers to estimate how explanatory variables influence the probability of land conversion and to compare the relative importance of different predictors. For this reason, it has been widely adopted as a benchmark model in studies of urban growth and land-use change [17–19].

However, the strengths of logistic regression are accompanied by important limitations. Although it is effective for estimating variable effects and interpreting how predictors influence the probability of land-use conversion, conventional regression models generally treat observations as independent units and do not explicitly model spatial dependence or autocorrelation [20–22].This limitation is especially important in corridor-specific urban transformation, where change is shaped not only by local node-level attributes but also by adjacency, connectivity, and relational dependence across the street network. Therefore, logistic regression is best treated here as a strong interpretable benchmark rather than a complete model of highly relational urban processes. Because of these limitations, recent urban research has increasingly adopted graph-based approaches, including Graph Neural Networks, which are better suited to learning from connected spatial structures.

### 2.3. Graph Neural Networks (GNN)

GNNs are designed for graph-structured data and can learn simultaneously from node attributes and network topology, this makes them particularly suitable for urban systems, where the significance of a location depends not only on its own characteristics but also on its position within a wider network [23,24]. This shift is especially relevant for residential-to-commercial corridor transformation. The issue is not only whether an individual node changes function, but also whether the predicted pattern reproduces the observed continuity of a corridor. Graph-based models make it possible to combine conventional predictors, such as centrality measures, with the adjacency structure of the street network itself. As a result, they are better suited to representing inter-node dependence and spatial continuity than conventional regression models, which may still produce fragmented predictions even when their predictors are statistically significant [24,25].

In summary, the literature indicates that commercial activity tends to concentrate on more accessible and better-connected streets and that the underlying processes of change are spatially dependent rather than isolated. It also suggests that, although logistic regression remains a valuable benchmark, graph-based models are more consistent with the relational nature of street-network processes [23,25,26]. What remains underdeveloped, however, is a corridor-scale, historically grounded explanation of why one corridor transformed commercially while nearby streets did not.

### 2.4. Research gap

The Previous studies have established that street-network accessibility and centrality influence pedestrian movement, commercial concentration, retail location, and land-use intensity [8,10–15]. However, most of this research has examined citywide or district-scale spatial relationships using contemporary or cross-sectional data, providing limited explanation of why commercial transformation develops along one specific corridor while nearby streets with comparable physical characteristics remain predominantly residential. Conventional land-use models, particularly Logistic Regression, offer interpretable estimates of how individual variables affect the probability of urban transformation [18–20], but they do not explicitly learn from the adjacency, connectivity, and relational dependence embedded within street networks [21–23]. Graph-based models can address this limitation by combining node-level attributes with network topology; nevertheless, their application to historically grounded, corridor-specific residential-to-commercial transformation remains insufficiently investigated. Accordingly, the research gap is threefold: limited attention has been given to the historical network conditions preceding corridor transformation; few studies have directly compared conventional statistical and graph-based models using identical street-network predictors; and previous evaluations have largely emphasized node-level classification accuracy while overlooking whether predicted changes reproduce the connected spatial structure of the observed corridor. To address these gaps, this study reconstructs Erbil’s 2004 street network and compares Logistic Regression with a Graph Convolutional Network to explain the residential-to-commercial transformation observed between 2004 and 2024, using both conventional classification metrics and spatial-coherence indicators.

## 3. Methodology

This study adopts a retrospective explanatory–predictive design to examine why a single corridor in Erbil transformed from predominantly residential use to commercial use between 2004 and 2024 while adjacent streets did not. The methodology combines historical spatial reconstruction, street-network centrality analysis, conventional statistical modeling, and graph-based learning so that both attribute effects and relational network effects can be evaluated within the same analytical framework.

### 3.1. Study design and temporal framework

The analytical logic is temporal. A case-study plan representing the study area in 2004 was reconstructed using historical spatial references. Google Earth Pro was consulted solely as a historical visual reference for identifying the street configuration existing in 2004; no Google Earth, Google Maps, or Google Street View imagery is reproduced in any figure in this manuscript.

### 3.2. Data preparation and variable definition

The working geospatial dataset was prepared from the 2004 case study plan, which reconstructed the historical street structure of the study area at a time when the area was still predominantly residential. The original street network was first represented as street edges, and these edges were then converted into nodes using QGIS 3.40 Bratislava. Each node in the resulting point-based layer represented a location derived from the 2004 street network and served as the basic observation unit for subsequent analysis.

All node coordinates were projected to EPSG:32638 (WGS 84/ UTM zone 38N), a projected coordinate reference system with units in meters based on the Universal Transverse Mercator method. This ensured that all distance-based operations were performed consistently in metric units. The dependent variable was defined as a binary land-use outcome based on the observed condition in 2024. Nodes representing locations along the corridor that had transformed into commercial use were assigned a value of 1, whereas nodes that remained residential were assigned a value of 0. S1 Fig, presents the case-study area.

The 2004 explanatory network was reconstructed by digitizing the historical street configuration identified from the case-study plan and historical reference imagery. Google Earth Pro was used only as a visual reference during this reconstruction process and no Google imagery is reproduced in the manuscript or Supporting Information. The resulting street centerlines were subsequently cleaned and processed in QGIS 3.40 Bratislava. The cleaned edges were converted into nodes, and only valid point geometries were retained for analysis. The attribute fields used in modeling were then checked so that Label and Weight were numeric, while rows with invalid labels were excluded. The binary outcome was assigned from the observed 2024 land-use condition: nodes located on the corridor that had converted to commercial use were coded 1, whereas nodes on adjoining streets that remained predominantly residential were coded 0. After filtering, the final analytical dataset contained 279 valid nodes, which formed the common observation base for both the Logistic Regression and GCN models.

In addition to the binary target variable, each node retained a weight attribute derived from the street width in the prepared spatial dataset in order to preserve local spatial importance. In this study, street width was classified into three ordinal weight levels: streets with a width of 10 m were assigned a weight of 1, streets with a width of 15 m were assigned a weight of 2, and streets with a width of 30 m were assigned a weight of 3. This weighting scheme assumes that wider streets have greater movement capacity, frontage visibility, and commercial exposure within the corridor structure. Ordinal weights were preferred to raw width values because they preserve the local street hierarchy while avoiding an unnecessarily strong scale effect from absolute width magnitudes in a relatively small case-study network. After the network centrality measures were computed, each centrality value was multiplied by the corresponding node weight so that the final predictors reflected both the structural position of the node in the network and its local spatial significance.

The analysis was implemented in Python using libraries aligned with each stage of the workflow. GeoPandas supported spatial data handling and coordinate transformation, while Pandas and NumPy were used for data preparation, weighting, feature construction, and z-score standardization. NetworkX was used to construct the graph and compute degree, betweenness, closeness, PageRank, straightness, and eigenvector centrality. Scikit-learn supported k-nearest-neighbor graph construction, the stratified 70:30 train–test split, logistic regression estimation, and performance evaluation using accuracy, precision, recall, F1-score, Jaccard similarity (IoU), ROC–AUC, the confusion matrix, and the classification report. The GCN model was implemented in PyTorch for tensor construction, model definition, optimization, loss computation, and probability estimation. Matplotlib was used for figures, and Pandas and GeoPandas were used to export tabular and spatial outputs. Because eigenvector centrality showed negligible explanatory contribution during preliminary feature screening, the final LR and GCN models were estimated using five predictors: degree, betweenness, closeness, PageRank, and straightness. The research code and minimal dataset supporting this study are publicly available in Zenodo at doi.org/10.5281/zenodo.21343914.

### 3.3. Street-network centrality measures

Following the logic of multiple centrality assessment, the combined set of measures captures complementary dimensions of the 2004 street system, including local connectivity, intermediary control, global accessibility, route efficiency, and recursive importance [27]. Six centrality measures were initially calculated because no single measure can fully represent the structural characteristics of a street network. Together, degree, betweenness, closeness, PageRank, straightness, and eigenvector centrality capture complementary dimensions of local connectivity, intermediary control, global accessibility, route directness, and recursive network importance [11,28]. Following preliminary feature screening, eigenvector centrality was excluded from the final models because it consistently showed the weakest explanatory and predictive contribution compared with the other measures. The remaining five predictors were retained to reduce unnecessary model complexity, preserve interpretability, and ensure that Logistic Regression and the GCN were evaluated using the same predictor set.

1. **Degree centrality** was used to represent **immediate local connectivity**, that is, how many direct links each node has to adjacent nodes. In street networks, it reflects local permeability and the number of immediate movement options around a node [27,28].


$$\begin{array}{c} C_{D}(i)=\sum_{j\in N(i)}a_{ij} \end{array}$$ (1)

where: $C_{D}(i)$ = degree of node i, $a_{ij}$= element of the adjacency matrix and N(i)= set of nodes

2. **Betweenness centrality** was used to capture **intermediary or corridor control**, since it identifies nodes that lie on many shortest paths between other nodes. This is especially relevant for commercial-corridor formation because highly traversed nodes tend to concentrate through-movement and exposure [27,29–31].


$$\begin{array}{c} C_{B}(i)=\sum_{s\text{?}i\text{?}t}\frac{\sigma_{st}(i)}{\sigma_{st}} \end{array}$$ (2)

where $C_{B}(i)$ is the betweenness centrality of node i, s and t are source and destination nodes, $\sigma_{st}$ denotes the number of shortest paths from s to t, and $\sigma_{st}(i)$ denotes the number of those shortest paths that pass through node i.

3. **Closeness centrality** was used to measure **global accessibility**, meaning how efficiently a node can reach all other nodes in the network. In urban analysis, this helps identify locations that are more centrally positioned within the overall street system [27,32,33].


$$\begin{array}{c} C_{C}(i)=\frac{N-\text{1}}{\sum_{j\text{?}i}d(i,j)} \end{array}$$ (3)

where: $C_{C}(i)$= closeness centrality of node i, N= total number of nodes in the network and $d_{ij}$= shortest-path distance between node i and node j

4. **PageRank centrality** was included to represent **recursive structural importance**, where a node is important not only because it has connections, but because it is connected to other important nodes. This adds a hierarchical dimension of network influence beyond simple connectivity [34–37].


$$\begin{array}{c} PR(i)=\frac{\text{1}-\alpha }{N}+\alpha \sum_{j\in N(i)}\frac{w_{ji}}{\sum_{k\in N(j)}w_{jk}}\;PR(j) \end{array}$$ (4)

where PR(i) is the PageRank of node i, alpha is the damping factor, N is the total number of nodes, N(i) is the set of nodes connected to node i, and $w_{ji}$ is the weight of the edge between nodes j and $w_{jk}$ is the sum of the weights of all edges connected from node j to its neighboring nodes and PR(j) is the PageRank value of node j.

5. **Straightness centrality** was used to evaluate **route directness**, that is, how closely movement through the network approximates straight-line travel. This is particularly suitable for street networks because commercial activity often favors routes that are not only connected, but also spatially efficient and legible [27,38].


$$\begin{array}{c} C_{S}(i)=\frac{\text{1}}{N-\text{1}}\sum_{j=\;\;/i}\frac{d_{E}(i,j)}{d_{G}(i,j)}\; \end{array}$$ (5)

Where: $C_{S}(i)$ represents the straightness centrality of node i. Nis the total number of nodes in the network. $d_{E}(i,j)$ refers to the Euclidean distance between nodes i and j, $d_{G}(i,j)$ represents the graph distance between nodes i and j.

After the centrality measures were computed on the reconstructed network, each centrality score was multiplied by the corresponding node weight so that the final predictors reflected both network position and local node significance [39–41]. The resulting weighted centrality variables were then standardized using z-score normalization before model training.


$$\begin{array}{c} x_{i}j=c_{i}j\times w_{i}\; \end{array}$$ (6)

where $c_{i}j$ is the raw centrality score and $w_{i}$ is the node-level street-width weight.

The weighted variables were subsequently standardized using z-score normalization based on the mean and standard deviation of the training subset, and the same scaling parameters were applied to the full feature set used in model training and evaluation.


$$\begin{array}{c} z_{ij}=\frac{x_{ij}-\mu_{j}^{\left(\text{train}\right)}}{\sigma_{j}^{\left(\text{train}\right)}}\; \end{array}$$ (7)

where $z_{ij}$ is the standardized weighted value of variable j for node i, $x_{ij}$ is the weighted feature value, $\mu_{j}^{\left(\text{train}\right)}$ is the mean of variable j in the training set, and $\sigma_{j}^{\left(\text{train}\right)}$ is the standard deviation of variable j in the training set. [42].

### 3.4. Feature scaling, main train–test split, and repeated validation design

To minimize information leakage, the dataset was divided into training and test subsets before final feature standardization. Preprocessing parameters were therefore estimated from the training data only and then transferred to the held-out test data. A stratified 70:30 train–test split was first adopted using a fixed random seed of 42. This main split was used for the primary model comparison and for producing the spatial prediction maps, ensuring direct comparability between Logistic Regression and the GCN.

For each split, the weighted centrality predictors were standardized using the mean and standard deviation of the training subset only. The same scaling parameters were then applied to the corresponding test subset. This procedure ensured that test nodes did not influence either scaling or class-weight estimation during model fitting, thereby reducing the risk of information leakage and overstated model performance.

In parallel, the graph supplied to the GCN was constructed as an undirected Euclidean k-nearest-neighbor network, with k capped at 8 and reduced automatically when the number of observations was smaller. Edge weights were defined by Euclidean distance; inverse distance was used where recursive centrality computation required it; and the GCN adjacency matrix was represented as a binary connectivity matrix with self-loops and symmetric normalization. The graph structure remained available in the transductive GCN setting, while the supervised loss was evaluated only on the training nodes.

To reduce dependence on a single train–test partition, an additional repeated-split robustness validation was conducted. The same stratified 70:30 split procedure was repeated across 30 random seeds. For each repetition, the training and test nodes were reselected while preserving the class distribution, training-only standardization was reapplied, and both the Logistic Regression and GCN models were re-estimated using the same five-predictor specification. The resulting classification and spatial-coherence metrics were summarized using the mean and standard deviation across the 30 repetitions.

### 3.5. Logistic Regression model

Logistic regression was adopted as the baseline statistical model because it is a standard and interpretable classifier for binary outcomes, making it well suited for benchmarking more complex learning models. Its coefficients provide a transparent description of the association between each predictor and the log-odds of commercial conversion, while predicted probabilities are obtained through the logistic response function [43–45]. To reduce the effect of class imbalance, weighted fitting was applied through balanced class weights, and the model was estimated with a maximum of 2,000 iterations. For node i, the probability of commercial conversion was modeled as


$$\begin{array}{c} P\left(y_{i}=\text{1}|x_{i}\right)=\frac{\text{1}}{\text{1}+\text{exp}\left(-\left(\beta_{\text{0}}+\sum_{k}\beta_{k}z_{ik}\right)\right)}\; \end{array}$$ (8)

where $P(y_{i}=\text{1}|x_{i})$ denotes the probability of commercial conversion for node i, $\beta_{\text{0}}$ is the intercept term, $\beta_{k}$ is the coefficient of predictor k, and $z_{ik}$ is the standardized value of predictor k for node i.

Logistic regression was used as an interpretable benchmark rather than as a complete spatial model. This choice was made because the study aims to evaluate whether graph-based learning adds explanatory and predictive value beyond a conventional binary land-use change model. Although spatial econometric models are appropriate for explicitly estimating spatial lag, spatial error, or spillover effects, they require a predefined spatial weights structure and primarily model spatial dependence through econometric parameters. In contrast, the present study focuses on a street network represented as a graph, where the relational structure among nodes is central to the research question. The GCN was therefore selected as the main relational model because it directly combines node-level predictors with adjacency-based learning. Spatial econometric modeling remains a valuable extension for future comparative work, but logistic regression provides a clearer and more widely used baseline for assessing the added value of graph-convolutional learning.

### 3.6. Graph convolutional network (GCN) model

A two-layer Graph Convolutional Network (GCN) was employed to examine whether the same centrality-derived predictors gained additional explanatory power when neighborhood effects were learned explicitly from the graph structure. In the final specification, each node was initialized with five standardized weighted predictors (degree_w, betw_w, close_w, pagerank_w, and straight_w). The graph was treated in a semi-supervised transductive setting: node embeddings were computed over the full k-nearest-neighbor graph, but the supervised loss was evaluated only on the training nodes using a Boolean training mask. Edge distances were used for distance-sensitive centrality calculations, whereas the GCN adjacency matrix was represented as a binary connectivity matrix with self-loops and symmetric normalization. The hidden layer contained 16 units with ReLU activation, the output layer contained 2 units for binary classification, optimization used Adam with a learning rate of 0.01, and training proceeded for 200 epochs. Class-weighted cross-entropy was used so that the minority commercial class exerted greater influence during fitting [46,47].

Accordingly, the first graph-convolution layer was defined as:


$$\begin{array}{c} H^{\left(\text{1}\right)}=ReLU\left(\hat{A}XW^{\left(\text{0}\right)}+b^{\left(\text{0}\right)}\right) \end{array}$$ (9)

The output layer was defined as:


$$\begin{array}{c} Z=\hat{A}H^{\left(\text{1}\right)}W^{\left(\text{1}\right)}+b^{\left(\text{1}\right)} \end{array}$$ (10)

In these expressions, Â denotes the symmetrically normalized adjacency matrix with self-loops, X is the input feature matrix, $W^{\left(\text{0}\right)}$ and $W^{\left(\text{1}\right)}$ are trainable weight matrices, and $b^{\left(\text{0}\right)}$ and $b^{\left(\text{1}\right)}$ are bias terms [46].

Node-level class probabilities were obtained by applying a row-wise SoftMax function:


$$\begin{array}{c} p_{i}=softmax\left(Z_{i}\right)\; \end{array}$$ (11)

The model was therefore estimated in a semi-supervised transductive setting, which is consistent with the original GCN formulation and preserves the common graph context for both training and test nodes while restricting supervision to the training subset [46,48] Optimization was based on a class-weighted cross-entropy loss evaluated over the training nodes only:


$$\begin{array}{c} L=-\left(\text{1}/|T|\right)\Sigma_{\left(i\in T\right)}w_{\left(y_{i}\right)}log\left(p_{\left(i,y_{i}\right)}\right)\; \end{array}$$ (12)

Here, T denotes the set of training nodes, $w_{\left(y_{i}\right)}$ is the weight assigned to the observed class of node i, and $p_{\left(i,y_{i}\right)}$ is the predicted probability of the true class. Optimization was performed using Adam [47].

During repeated-split validation, the same GCN architecture and hyperparameters were retained for each repetition, but the training and test masks were updated according to the current stratified split. The model was reinitialized and retrained for each random seed so that the reported robustness results reflected independent train–test partitions rather than repeated evaluation of a single trained model.

Feature screening was conducted before the final comparative estimation using three complementary indicators: the bivariate correlation between each weighted predictor and the commercial label, the corresponding Logistic Regression coefficient, and the GCN feature-contribution score derived from the trained model. Eigenvector centrality consistently showed the weakest contribution across these indicators, with a bivariate correlation of −0.073, a Logistic Regression coefficient of −0.153, and a GCN contribution score of 0.089. These results indicated that eigenvector centrality added limited discriminatory information beyond the other centrality measures in this corridor-scale network. Its exclusion therefore reduced redundancy and model complexity without removing a substantively important predictor. The final Logistic Regression and GCN models were consequently estimated using weighted degree, betweenness, closeness, PageRank, and straightness centrality.

### 3.7. Model evaluation

Model performance was evaluated at two complementary levels. First, conventional predictive accuracy was assessed on the held-out test set using five standard classification metrics: accuracy, precision, recall, F1-score, and the area under the receiver operating characteristic curve (ROC–AUC). These measures quantified the ability of the models to distinguish between residential and commercial nodes at the individual-node level. Precision was computed as TP/ (TP + FP), recall as TP/ (TP + FN), F1-score as 2 × (Precision × Recall)/ (Precision + Recall), and Intersection over Union (IoU) as TP/ (TP + FP + FN) [49–51].

While these conventional metrics evaluate classification performance at the node level, they do not indicate whether predicted commercial nodes form a spatial pattern consistent with the observed corridor structure. Therefore, a second level of evaluation was conducted to assess spatial coherence. This was done by extracting the subgraph composed of all nodes predicted as commercial and then examining its connected structure. Three aspects were considered: the number of connected components, the fragmentation of the predicted-positive pattern, and the proportion of predicted commercial nodes contained in the largest connected component.

Spatial coherence was then examined on the subgraph of predicted commercial nodes. Fragmentation [50–53] was computed as


$$\begin{array}{c} \text{F}=\text{1}-\frac{\sum_{\text{k}}{\text{s}}_{\text{k}}\left({\text{s}}_{\text{k}}-\text{1}\right)}{{\text{N}}_{\text{pos}}\left({\text{N}}_{\text{pos}}-\text{1}\right)}\; \end{array}$$ (13)

where F denotes fragmentation, ${\text{s}}_{\text{k}}$ is the number of predicted positive nodes in connected component k, and ${\text{N}}_{\text{pos}}$ is the total number of predicted positive nodes in the evaluated subgraph [54,55].The Largest Connected Component Ratio (LCCR) was computed as $LCCR=\frac{s_{max}}{N_{\text{pos}}}$, where $s_{\text{max}}$ is the size of the largest connected component and $N_{\text{pos}}$ is the total number of predicted positive nodes. Robustness was examined in two ways. First, a specification sensitivity analysis compared the original six-predictor model with the final five-predictor model. Second, repeated-split validation was used to examine whether the findings were sensitive to the particular random partition of the data. The stratified 70:30 split was repeated across 30 random seeds, and both models were retrained and re-evaluated for each repetition. Classification metrics were calculated on the held-out test nodes for each split and summarized as mean ± standard deviation. Spatial-coherence metrics were also recalculated on the held-out test nodes for each repetition to determine whether the corridor-continuity advantage of the GCN remained stable across alternative train–test partitions.

The stratified 70:30 split was repeated across 30 random seeds, and both models were retrained and re-evaluated for each repetition. Classification metrics were calculated on the held-out test nodes for each split and summarized as mean ± standard deviation. Spatial-coherence metrics were also recalculated on the held-out test nodes for each repetition to determine whether the corridor-continuity advantage of the GCN remained stable across alternative train–test partitions.

For the main split, spatial-coherence metrics were reported for both the held-out test nodes and the full dataset. The test-set results were used as validation evidence, whereas the full-dataset results were interpreted as descriptive spatial-pattern diagnostics showing how each model represented the complete corridor structure.

### 3.8. Analytical interpretation

The final interpretation proceeded at two complementary levels. At the explanatory level, the Logistic Regression coefficients and the comparative feature contributions of the GCN were used to identify which centrality dimensions were most strongly associated with commercial transformation. At the classification level, the two models were compared using conventional node-level metrics, including accuracy, precision, recall, F1-score, ROC–AUC, and IoU. These metrics assessed how well each model classified individual nodes as residential or commercial.

At the spatial level, the predicted commercial nodes were evaluated as a connected subgraph to determine whether each model reproduced the corridor-like structure of transformation. This distinction was important because a model may correctly classify many individual nodes while still producing a fragmented spatial pattern. Therefore, the interpretation focused not only on whether transformed nodes were identified, but also on whether the predicted pattern reproduced the spatial continuity of the observed commercial corridor.

The repeated-split validation was used to distinguish stable model behavior from results that may depend on one particular train–test partition. Accordingly, the GCN was interpreted as providing a methodological advantage only where its spatial-coherence performance remained stronger across repeated splits, rather than being treated as universally superior across all conventional classification metrics.

### 3.9. Methodological contribution

Methodologically, the approach treats the 2004 street network not as a static background map but as a prior structural condition capable of shaping later land-use change. By comparing a conventional statistical classifier with a graph-based relational model on the same historical predictors, the method tests whether explicit network dependence adds explanatory value to the study of corridor-specific transformation. This design makes it possible to distinguish between explanations based only on node-level accessibility and explanations that also depend on relational embedding within the wider street system. By adding repeated stratified validation and spatial-coherence diagnostics, the workflow evaluates both predictive reliability and the spatial structure of model outputs, which is especially important for corridor-based transformation.where continuity is part of the phenomenon being explained. The methodological workflow is summarized in S2 Fig.

## 4. Results

After data preparation, the street-network centrality measures were calculated for the reconstructed 2004 street network and used as predictors of the observed residential-to-commercial transformation in 2024. The final five-predictor specification included weighted degree centrality, weighted betweenness centrality, weighted closeness centrality, weighted PageRank centrality, and weighted straightness centrality. Eigenvector centrality was excluded from the final model because the preliminary feature-screening stage indicated that it had the weakest explanatory contribution S3 Fig (A -E).

The factor-comparison results show that the predictors did not contribute equally to commercial transformation (S3 Fig-F). Weighted straightness centrality showed the strongest and most consistent relationship with the commercial label, with a correlation of 0.630, a Logistic Regression coefficient of 2.896, and the highest GCN importance score of 10.789. This indicates that route directness was a particularly important structural condition in the emergence of the commercial corridor. Weighted degree centrality also showed a high GCN importance score of 8.233, although its Logistic Regression coefficient was negative. This difference suggests that some predictors may operate differently when treated as independent node-level variables compared with when they are incorporated within a graph-learning structure S4 Fig.

Using the main stratified 70:30 train–test split with random_state = 42, both models achieved acceptable to strong classification performance. Logistic Regression achieved an accuracy of 0.857, precision of 0.636, recall of 1.000, F1-score of 0.778, and ROC–AUC of 0.966. The GCN achieved an accuracy of 0.881, precision of 0.690, recall of 0.952, F1-score of 0.800, and ROC–AUC of 0.952. In this main split, therefore, the GCN performed better in accuracy, precision, and F1-score, whereas Logistic Regression retained slightly higher recall and ROC–AUC Table 1.

**Table 1:** Classification performance of Logistic Regression and GCN models on the main 70:30 split.

Model	Accuracy	Precision	Recall	F1	ROC–AUC
Logistic Regression	0.857	0.636	1.000	0.778	0.966
GCN	0.881	0.690	0.952	0.800	0.952

In addition to conventional classification performance, spatial-coherence metrics were used to assess whether the predicted commercial nodes formed a continuous corridor-like pattern. On the held-out test set, the GCN produced fewer connected components than Logistic Regression, decreasing from 14 to 8 components. Fragmentation also declined from 0.920 in Logistic Regression to 0.852 in the GCN, while the largest connected component ratio increased from 0.212 to 0.276. IoU improved from 0.636 to 0.667, and the positive-class F1-score increased from 0.778 to 0.800 Table 2.

**Table 2:** Spatial coherence comparison of Logistic Regression and GCN predictions.

Scope	Model	Evaluated nodes	Predicted positive nodes	Connected components	Fragmentation	LCCR	IoU	F1
Test only	Logistic Regression	84	33	14	0.920	0.212	0.636	0.778
Test only	GCN	84	29	8	0.852	0.276	0.667	0.800
Full dataset	Logistic Regression	279	122	5	0.535	0.648	0.566	0.723
Full dataset	GCN	279	96	3	0.246	0.865	0.684	0.812

The same tendency was more pronounced in the full-dataset comparison. Logistic Regression predicted 122 commercial nodes distributed across five connected components, with fragmentation of 0.535 and an LCCR of 0.648. By contrast, the GCN predicted 96 commercial nodes distributed across only three connected components, with substantially lower fragmentation of 0.246 and a higher LCCR of 0.865. The GCN also improved IoU from 0.566 to 0.684 and positive-class F1-score from 0.723 to 0.812. These full-dataset results should be interpreted as descriptive spatial-pattern diagnostics rather than as independent validation results; nevertheless, they are useful for evaluating how each model represents the complete corridor structure.

To reduce dependence on a single train–test partition, a repeated-split robustness validation was conducted. The stratified 70:30 split was repeated across 30 random seeds. For each repetition, the same five-predictor feature set, training-only standardization procedure, Logistic Regression specification, and GCN architecture were used. Classification metrics were calculated on the held-out test nodes for each split and summarized using the mean and standard deviation.

The repeated-split classification results show that the difference between the two models is more nuanced than the main split alone suggests. Across 30 splits, the GCN achieved slightly higher mean accuracy and precision, with an accuracy of 0.825 ± 0.033 and precision of 0.611 ± 0.054. Logistic Regression achieved an accuracy of 0.822 ± 0.031 and precision of 0.589 ± 0.042. However, Logistic Regression achieved higher recall, F1-score, and ROC–AUC, with recall of 0.981 ± 0.046, F1-score of 0.735 ± 0.035, and ROC–AUC of 0.922 ± 0.020. The GCN achieved recall of 0.852 ± 0.097, F1-score of 0.708 ± 0.050, and ROC–AUC of 0.914 ± 0.024 Table 3.

**Table 3:** Repeated stratified 70:30 classification validation across 30 random splits.

Model	Accuracy	Precision	Recall	F1	ROC–AUC
GCN	0.825 ± 0.033	0.611 ± 0.054	0.852 ± 0.097	0.708 ± 0.050	0.914 ± 0.024
Logistic Regression	0.822 ± 0.031	0.589 ± 0.042	0.981 ± 0.046	0.735 ± 0.035	0.922 ± 0.020

These repeated-split results indicate that the GCN should not be interpreted as universally superior across all conventional node-level classification metrics. Rather, the GCN remained competitive in overall classification performance, while Logistic Regression remained stronger in recall and slightly stronger in average F1-score and ROC–AUC. The higher recall of Logistic Regression indicates that it was more inclusive in identifying transformed nodes. However, this broader sensitivity was accompanied by a tendency to predict a larger and more spatially dispersed set of commercial nodes.

The repeated spatial-coherence validation provides additional support for the central argument of the study. Across 30 held-out test splits, the GCN predicted fewer positive nodes on average than Logistic Regression, with 29.533 ± 4.462 predicted commercial nodes compared with 35.133 ± 3.137 for Logistic Regression. More importantly, the GCN produced fewer connected components, with 11.467 ± 1.717 components compared with 16.167 ± 2.001 for Logistic Regression. Fragmentation was also lower for the GCN, with 0.898 ± 0.051 compared with 0.934 ± 0.020 for Logistic Regression. The GCN further achieved a higher LCCR, with 0.222 ± 0.091 compared with 0.176 ± 0.049 for Logistic Regression.

Although Logistic Regression achieved higher average IoU and F1-score across the repeated test splits, the GCN consistently produced a more graph-topologically coherent prediction pattern. Its lower number of connected components, lower fragmentation, and higher LCCR indicate that its predicted commercial nodes were less dispersed and more likely to form a continuous corridor-like structure. Therefore, the repeated validation refines rather than overturns the main finding: the principal advantage of the GCN lies not in universal superiority across all classification metrics, but in its stronger ability to reproduce the spatial continuity of corridor-based commercial transformation Table 4.

**Table 4:** Repeated spatial-coherence validation on held-out test nodes across 30 random splits.

Model	Predicted positive nodes	Connected components	Fragmentation	LCCR	IoU	F1
GCN	29.533 ± 4.462	11.467 ± 1.717	0.898 ± 0.051	0.222 ± 0.091	0.550 ± 0.059	0.708 ± 0.050
Logistic Regression	35.133 ± 3.137	16.167 ± 2.001	0.934 ± 0.020	0.176 ± 0.049	0.582 ± 0.044	0.735 ± 0.035

Overall, the results show that both Logistic Regression and the GCN provided acceptable to strong evidence for modeling residential-to-commercial transformation. However, the two models emphasized different dimensions of performance. Logistic Regression showed higher sensitivity to transformed nodes, especially across repeated validation, while the GCN provided a more spatially coherent representation of the commercial corridor. This distinction is central to the study because the research question is not only whether individual transformed nodes can be classified correctly, but whether the model can reproduce the corridor-based structure through which urban transformation actually occurred.

## 5. Discussion

The findings demonstrate that the reconstructed 2004 street-network structure played a meaningful role in explaining the corridor-based residential-to-commercial transformation observed in 2024. The results support the central argument that commercial conversion in the study area was not only associated with individual node-level centrality values, but also with the relational structure of the street network. This is important because the transformation did not occur as a random distribution of isolated commercial nodes; rather, it emerged as a corridor-like pattern shaped by accessibility, route directness, and spatial connectivity.

The preliminary feature-screening results showed that the centrality measures did not contribute equally to prediction. Eigenvector centrality had the weakest contribution and was therefore excluded from the final comparative models. This suggests that recursive prestige within the wider network was less informative in this corridor-scale case than measures more directly related to accessibility, passage, and route efficiency. By contrast, weighted straightness centrality showed the strongest and most consistent association with commercial transformation. This finding indicates that route directness was a particularly important structural condition in the emergence of the commercial corridor. In practical terms, streets that provided more direct movement paths were more likely to support commercial conversion because they offered greater visibility, accessibility, and movement efficiency.

The classification results show that both Logistic Regression and the GCN were capable of modeling residential-to-commercial transformation with acceptable to strong predictive performance. However, the repeated-split validation clarified that the difference between the two models should not be interpreted as a simple overall superiority of the GCN. In the main 70:30 split, the GCN achieved higher accuracy, precision, and F1-score, while Logistic Regression retained slightly higher recall and ROC–AUC. Across the 30 repeated stratified splits, the GCN remained slightly stronger in mean accuracy and precision, whereas Logistic Regression achieved higher recall, F1-score, and ROC–AUC. This means that Logistic Regression was more sensitive in identifying transformed nodes, but it also tended to classify a larger number of nodes as commercial.

This distinction is important for interpreting the two models. Logistic Regression functions as a strong and interpretable baseline because it evaluates how each predictor contributes to the probability of commercial transformation. Its high recall indicates that it was effective in capturing most transformed nodes. However, this broader sensitivity also produced a more spatially dispersed prediction pattern. In other words, Logistic Regression was more inclusive, but less selective in reproducing the corridor structure. This reflects a limitation of conventional node-level classification: even when the predictors are derived from network centrality, the model still treats observations primarily as individual units rather than as embedded parts of a connected spatial system.

The GCN, in contrast, incorporated graph connectivity directly into the learning process. Its main advantage therefore appeared not as universal superiority across all conventional classification metrics, but as a stronger ability to reproduce graph-topological spatial coherence. In both the main split and the repeated spatial-coherence validation, the GCN produced fewer connected components, lower fragmentation, and a higher largest connected component ratio than Logistic Regression. These results indicate that the GCN predictions were less scattered and more likely to form a continuous corridor-like structure. This is central to the research question because the objective was not only to classify transformed nodes correctly, but also to explain why transformation appeared as a coherent corridor rather than as isolated commercial conversions.

The full-dataset spatial diagnostics further support this interpretation. While these results should not be treated as independent validation, they are useful for examining how each model represents the complete spatial structure of the study area. Logistic Regression predicted a larger number of commercial nodes, but these were distributed across more fragmented spatial components. The GCN predicted fewer commercial nodes, yet these predictions were more concentrated and spatially continuous. This suggests that the GCN was less prone to extending commercial prediction into isolated residential locations and more effective in identifying the main transformation corridor as a coherent spatial entity.

From a practical planning perspective, the proposed framework can function as an early decision-support tool for identifying streets that may experience increasing pressure for residential-to-commercial conversion. By combining historical street-network characteristics with spatially coherent model predictions, planning authorities could identify potential transformation corridors before land-use change becomes extensive. This information may support proactive corridor planning, zoning review, infrastructure investment, traffic and parking management, pedestrian improvements, and the provision of public services. It may also help planners anticipate conflicts between residential and commercial activities and introduce appropriate controls concerning building use, access, loading, signage, noise, and public-space requirements. The model should therefore be understood as a screening and planning-support framework rather than as a deterministic prediction of future land-use change.

The framework is also potentially adaptable to other cities because it uses widely available street-network data, graph representations, and transferable centrality measures. However, direct application without local adjustment would not be appropriate because patterns of commercial transformation are influenced by urban morphology, street hierarchy, transport systems, planning regulations, land-market conditions, and socioeconomic characteristics. Application in another city would therefore require reconstruction of the relevant historical network, local definition of the transformation outcome, recalibration of street-width weights and graph-construction parameters, and retraining and validation of both models using local data. Future multi-city testing would help determine which findings are transferable across urban contexts and which remain specific to the Erbil corridor.

The findings also contribute to the broader literature on street centrality and land-use transformation. Previous studies have shown that commercial activities tend to concentrate along streets with stronger accessibility and movement potential. This study extends that literature by showing that, at the corridor scale, the spatial arrangement of predicted transformation is itself an important dimension of model performance. The results suggest that graph-based learning is especially useful when the research problem involves spatial dependence, adjacency, and corridor continuity rather than only pointwise classification accuracy.

At the same time, the findings should be interpreted within several limitations. First, the study focuses on a single corridor in Erbil, and the results should therefore be understood as case-specific rather than universally generalizable. Second, the 2004 street network was reconstructed from historical spatial sources, which introduces some uncertainty despite careful digitization and cleaning. Third, the dependent variable was represented as a binary residential/commercial classification, while real land-use transformation may involve mixed-use, gradual conversion, and different intensities of commercial activity. Fourth, although repeated-split validation strengthens the robustness of the findings, future research should test the approach across additional corridors, neighborhoods, and cities. Finally, future work could compare the GCN with spatial econometric models, random forest, gradient boosting, and alternative graph neural network architectures to further clarify the conditions under which graph-based learning provides the greatest advantage.

Overall, the Discussion supports a refined conclusion: the GCN should not be presented as superior across all predictive metrics. Instead, its principal value lies in its ability to capture the spatial coherence of corridor-based transformation. Logistic Regression remains valuable as an interpretable and sensitive benchmark, while the GCN adds a relational modeling capacity that is better aligned with the connected nature of street-network processes. This distinction strengthens the methodological contribution of the study by showing that evaluating urban transformation models requires not only conventional classification metrics, but also spatial-coherence measures that reflect how urban change actually unfolds.

## Supporting information

S1 Data Minimal dataset. (ZIP)

S1 Fig Study area in Erbil, Iraq. (DOCX)

S2 Fig Research Methodology. (DOCX)

S3 Fig Street-network centrality measures and feature importance. (DOCX)

S4 Fig Predicted spatial pattern of commercial land-use transformation based on calculated centrality values. (DOCX)
