# Supplementary material for: Graph convolutional network for modeling corridor-based urban transformation: Revealing spatial coherence in land-use change in Erbil
Source: PLoS One. 2026 Aug 27;21(8):e0357090. doi: 10.1371/journal.pone.0357090 (PMC13521350; doi:10.1371/journal.pone.0357090)
Supplement: Supplementary file 1 [file pone.0357090.s002.docx]

|  |  |
| --- | --- |

**S1 Fig. Study area in Erbil, Iraq.**

**(A)** the location of the study area within Erbil. **(B)** detailed view of the selected corridor used for the analysis.

**Contains information from OpenStreetMap and OpenStreetMap Foundation, which is made available under the Open Database License.**
