# Supplementary material for: Graph convolutional network for modeling corridor-based urban transformation: Revealing spatial coherence in land-use change in Erbil
Source: PLoS One. 2026 Aug 27;21(8):e0357090. doi: 10.1371/journal.pone.0357090 (PMC13521350; doi:10.1371/journal.pone.0357090)
Supplement: Supplementary file 2 [file pone.0357090.s003.docx]

**S2 Fig. Research Methodology.**

Workflow illustrating data preparation, graph construction, calculation of street-network centrality measures, feature scaling, model development using Logistic Regression and Graph Convolutional Network (GCN), model evaluation, and interpretation of the results.
