# Supplementary material for: Graph convolutional network for modeling corridor-based urban transformation: Revealing spatial coherence in land-use change in Erbil
Source: PLoS One. 2026 Aug 27;21(8):e0357090. doi: 10.1371/journal.pone.0357090 (PMC13521350; doi:10.1371/journal.pone.0357090)
Supplement: Supplementary file 3 [file pone.0357090.s004.docx]

|  |  |
| --- | --- |
| A. Degree Centrality | B. Betweenness Centrality |
|  |  |
| C. Closeness Centrality | D. PageRank Centrality |
|  |  |
| E. Straightness Centrality | F. Feature Importance for Commercial prediction |

**S3 Fig. Street-network centrality measures and feature importance.**

**(A)** Degree centrality. **(B)** Betweenness centrality. **(C)** Closeness centrality.  **(D)** PageRank centrality.  **(E)** Straightness centrality.  **(F)** feature importance for commercial land-use prediction.

**Contains information from OpenStreetMap and OpenStreetMap Foundation, which is made available under the Open Database License.**
