# Supplementary material for: Graph convolutional network for modeling corridor-based urban transformation: Revealing spatial coherence in land-use change in Erbil
Source: PLoS One. 2026 Aug 27;21(8):e0357090. doi: 10.1371/journal.pone.0357090 (PMC13521350; doi:10.1371/journal.pone.0357090)
Supplement: Supplementary file 4 [file pone.0357090.s005.docx]

|  |  |
| --- | --- |
| A. GCN Prediction | B. Logistic Regression Prediction |

**S4 Fig.** **Predicted spatial pattern of commercial land-use transformation based on calculated centrality values**.

**(A)** the highest likelihood of conversion from residential use in the initial stage to commercial use by GCN Predictions. **(B)** the highest likelihood of conversion from residential use in the initial stage to commercial use by Logistic Regression Prediction.

**Contains information from OpenStreetMap and OpenStreetMap Foundation, which is made available under the Open Database License.**
