# Supplementary material for: Graph convolutional network for modeling corridor-based urban transformation: Revealing spatial coherence in land-use change in Erbil
Source: PLoS One. 2026 Aug 27;21(8):e0357090. doi: 10.1371/journal.pone.0357090 (PMC13521350; doi:10.1371/journal.pone.0357090)
Supplement: Supplementary file 5 [file pone.0357090.s007.docx]

Journal Requirements:

1. Please ensure that your manuscript meets PLOS ONE's style requirements, including those for file naming. The PLOS ONE style templates can be found at
   [https://journals.plos.org/plosone/s/file?id=wjVg/PLOSOne_formatting_sample_main_body.pdf](https://track.editorialmanager.com/CL0/https:%2F%2Fjournals.plos.org%2Fplosone%2Fs%2Ffile%3Fid=wjVg%2FPLOSOne_formatting_sample_main_body.pdf/1/010f019f4a0f4988-56150d00-3ad7-4abc-9067-3da122708715-000000/mWiDJ9RYId-AYfq8c5eXSFcXS7N1zTRJbBkuVxuK-Mk=258) and [https://journals.plos.org/plosone/s/file?id=ba62/PLOSOne_formatting_sample_title_authors_affiliations.pdf](https://track.editorialmanager.com/CL0/https:%2F%2Fjournals.plos.org%2Fplosone%2Fs%2Ffile%3Fid=ba62%2FPLOSOne_formatting_sample_title_authors_affiliations.pdf/1/010f019f4a0f4988-56150d00-3ad7-4abc-9067-3da122708715-000000/aaD9JowCu-82p8mOBiXBANEze4cJ1npeFkVMd8s3XkU=258)

Thank you for bringing this to our attention. We have carefully reviewed the manuscript and revised it to comply with the PLOS ONE formatting and style requirements, including the required file naming conventions. The revised files have been prepared according to the journal's formatting guidelines.

2. Please note that PLOS One has specific guidelines on code sharing for submissions in which author-generated code underpins the findings in the manuscript. In these cases, we expect all author-generated code to be made available without restrictions upon publication of the work. Please review our guidelines at <https://journals.plos.org/plosone/s/materials-and-software-sharing#loc-sharing-code> and ensure that your code is shared in a way that follows best practice and facilitates reproducibility and reuse.

Thank you for this requirement. We have reviewed the PLOS ONE code-sharing policy. The Python code developed for data preprocessing, graph construction, model implementation, and evaluation has already been uploaded to Zenodo, and a DOI has been reserved (DOI: 10.5281/zenodo.21343914). The repository will remain private during the peer-review process and will be made publicly available after the manuscript receives final acceptance, in accordance with the journal's code-sharing policy.

3. Please provide a complete Data Availability Statement in the submission form, ensuring you include all necessary access information or a reason for why you are unable to make your data freely accessible. If your research concerns only data provided within your submission, please write "All data are in the manuscript and/or supporting information files" as your Data Availability Statement.

 The processed dataset and the source code supporting the findings of this study have been deposited in Zenodo (DOI: 10.5281/zenodo.21343914). The repository is currently private during peer review and will be made publicly available upon final acceptance of the manuscript, in accordance with the journal's data and code sharing policy.

4. Please include a separate caption for each figure in your manuscript.

 Thank you for this comment. We have carefully reviewed all figures and ensured that each figure has a separate, complete, and descriptive caption in accordance with the PLOS ONE formatting guidelines. The figure captions have been revised where necessary to improve clarity and comply with the journal's requirements

5. [We note that Figure(s) 1, 3, 4 in your submission contain [map/satellite] images which may be copyrighted. All PLOS content is published under the Creative Commons Attribution License (CC BY 4.0), which means that the manuscript, images, and Supporting Information files will be freely available online, and any third party is permitted to access, download, copy, distribute, and use these materials in any way, even commercially, with proper attribution. For these reasons, we cannot publish previously copyrighted maps or satellite images created using proprietary data, such as Google software (Google Maps, Street View, and Earth). For more information, see our copyright guidelines: http://journals.plos.org/plosone/s/licenses-and-copyright.](We%20note%20that%20Figure(s)%201,%203,%204%20in%20your%20submission%20contain%20%5bmap/satellite%5d%20images%20which%20may%20be%20copyrighted.%20All%20PLOS%20content%20is%20published%20under%20the%20Creative%20Commons%20Attribution%20License%20(CC%20BY%204.0),%20which%20means%20that%20the%20manuscript,%20images,%20and%20Supporting%20Information%20files%20will%20be%20freely%20available%20online,%20and%20any%20third%20party%20is%20permitted%20to%20access,%20download,%20copy,%20distribute,%20and%20use%20these%20materials%20in%20any%20way,%20even%20commercially,%20with%20proper%20attribution.%20For%20these%20reasons,%20we%20cannot%20publish%20previously%20copyrighted%20maps%20or%20satellite%20images%20created%20using%20proprietary%20data,%20such%20as%20Google%20software%20(Google%20Maps,%20Street%20View,%20and%20Earth).%20For%20more%20information,%20see%20our%20copyright%20guidelines:%20http://journals.plos.org/plosone/s/licenses-and-copyright.We%20require%20you%20to%20either%20(1)%20present%20written%20permission%20from%20the%20copyright%20holder%20to%20publish%20these%20figures%20specifically%20under%20the%20CC%20BY%204.0%20license,%20or%20(2)%20remove%20the%20figures%20from%20your%20submission:a.%20You%20may%20seek%20permission%20from%20the%20original%20copyright%20holder%20of%20Figure(s)%201,%203,%204%20to%20publish%20the%20content%20specifically%20under%20the%20CC%20BY%204.0%20license.We%20recommend%20that%20you%20contact%20the%20original%20copyright%20holder%20with%20the%20Content%20Permission%20Form%20(http://journals.plos.org/plosone/s/file?id=7c09/content-permission-form.pdf)%20and%20the%20following%20text:#Natural Earth (public domain): http://www.naturalearthdata.com/)

Thank you for bringing this issue to our attention. We have carefully reviewed the figures. Figure 1 has been revised to remove the Google Earth imagery and ensure compliance with the PLOS ONE copyright and licensing requirements. Figures 3 and 4 were originally created in QGIS using OpenStreetMap data and do not contain any Google Earth or Google Maps imagery. Therefore, no changes to these figures were necessary. Appropriate attribution to OpenStreetMap has been included in the figure captions.

6. We note you have included a table to which you do not refer in the text of your manuscript. Please ensure that you refer to Table 1, 2, 3, 4 in your text; if accepted, production will need this reference to link the reader to the Table.

 Thank you for this comment. We have reviewed the tables and revised their formatting to comply with the PLOS ONE guidelines. All tables are presented as editable tables with separate, descriptive titles and have been placed in the manuscript immediately after their first citation in the text, in accordance with the journal's formatting requirements.

7. If the reviewer comments include a recommendation to cite specific previously published works, please review and evaluate these publications to determine whether they are relevant and should be cited. There is no requirement to cite these works unless the editor has indicated otherwise.

Thank you for this comment. We carefully reviewed all references suggested by the reviewers and evaluated their relevance to the present study. Relevant references have been incorporated into the revised manuscript where appropriate.

8. Please review your reference list to ensure that it is complete and correct. If you have cited papers that have been retracted, please include the rationale for doing so in the manuscript text, or remove these references and replace them with relevant current references. Any changes to the reference list should be mentioned in the rebuttal letter that accompanies your revised manuscript. If you need to cite a retracted article, indicate the article’s retracted status in the References list and also include a citation and full reference for the retraction notice

Thank you for this comment. We have carefully reviewed the reference list to ensure that it is complete, accurate, and up to date. The references have been checked for correctness, and any necessary revisions have been made. We also confirmed that none of the cited references have been retracted.

Additional Editor Comments :

1. Strengthen the Introduction and Literature Review:
   Please substantially expand the introduction section. In the literature review, avoid merely listing previous studies; instead, focus on a comprehensive review that deeply analyzes the logical connections between studies. Please be sure to define the "research gap" more clearly, detailing the unresolved issues in existing research and the unique contributions of this study.

Thank you for this valuable comment. We have substantially revised both the **Introduction** and the **Literature Review**. The Introduction has been expanded to provide a stronger background, clearly state the study objectives, and highlight the novelty and contributions of the research. The Literature Review has been reorganized into thematic sections that critically synthesize previous studies rather than listing them individually. In addition, we added a dedicated **Research Gap** section that clearly identifies the unresolved issues in the literature and explains how the present study addresses these gaps through the comparison of Logistic Regression and Graph Convolutional Networks and the incorporation of spatial coherence as an additional evaluation criterion.

2. Optimize the Methodology Description:
   The methodology section is generally clear, but it is recommended to streamline the repetitive descriptions regarding model training and data preparation. In addition, please provide further explanation for excluding "eigenvector centrality" from the final model. Even if its contribution is relatively small, providing clear statistical or theoretical justification will enhance the persuasiveness of the model.

Thank you for this valuable comment. We have revised the Methodology section to improve clarity and reduce repetitive descriptions related to data preparation and model training. In addition, we have expanded the explanation for excluding eigenvector centrality, providing both theoretical and empirical justification. Specifically, eigenvector centrality exhibited weak discriminative power in the study corridor and showed limited contribution to model performance, making its inclusion unnecessary while improving model interpretability.

3. Deepen the Results and Discussion:
   Please expand the discussion on "practical implications" in the discussion section. For example, how can planners use this model to assist future land-use decisions? Is this framework applicable to other cities with drastically different street layouts? Addressing these points will significantly enhance the practical value of the research.

Thank you for this valuable comment. We have expanded the Discussion section by adding a dedicated discussion on the practical implications of the proposed framework. The revised manuscript now explains how the model can support urban planners in anticipating corridor-based commercial transformation, informing zoning and infrastructure decisions, and guiding sustainable land-use management. We also discuss the transferability of the proposed framework to other cities, highlighting its general applicability while acknowledging the need for local calibration to account for differences in urban morphology, planning policies, and socio-economic conditions.

4. Figures, Tables, and Formatting Revisions:
   Please check and increase the font size in the figures and tables to ensure that all labels are clear and readable. At the same time, please conduct a final comprehensive review of the reference format to ensure consistency in capitalization and citation style.

Thank you for this valuable comment. We have reviewed all figures and tables and increased the font sizes where necessary to improve the readability of labels, legends, and annotations. In addition, we conducted a comprehensive review of the reference list and in-text citations to ensure consistency with the journal's formatting requirements, including capitalization, citation style, and reference formatting throughout the manuscript.

5. Data Availability:
   Please clearly provide the repository link or DOI for the supporting code within the manuscript to ensure the reproducibility of the research.

Thank you for this comment. We have revised the manuscript to include the Zenodo repository DOI for the supporting code to facilitate reproducibility. The source code has been deposited in Zenodo (DOI: 10.5281/zenodo.21343914). The repository is currently under embargo and will be made publicly accessible upon final acceptance of the manuscript.

Review Comments to the Author

Reviewer #1:

1. Introduction and Research Gap

The introduction is a bit short and should be fleshed out further.

Also, the literature review reads more like a summary of previous studies than a discussion of how those studies relate to one another. I would suggest making this section more cohesive by synthesizing the literature rather than listing individual papers.

The research gap is mentioned, but it could be emphasized more clearly. I would recommend explaining in greater detail what existing studies have not addressed and how this work builds upon or differs from them.

Thank you for this valuable comment. We have substantially revised both the **Introduction** and the **Literature Review**. The Introduction has been expanded to provide a stronger background, clearly define the study objectives, and emphasize the novelty and contributions of the research. The Literature Review has been reorganized into thematic sections that critically synthesize previous studies rather than simply summarizing individual works. In addition, a dedicated **Research Gap** section has been added to clearly identify the unresolved issues in the existing literature and explain how the present study addresses these gaps through historical street-network reconstruction, the comparison of Logistic Regression and Graph Convolutional Networks, and the incorporation of spatial coherence as an additional model evaluation criterion.

2. Methodology

The methodology is generally well described and easy to understand.

That said, some implementation details are repeated throughout the section. For example, the discussion of the model training process and data preparation could be condensed without affecting the reader's understanding.

In addition, the decision to exclude eigenvector centrality from the final model could be explained in a little more detail. Although the paper mentions that it contributed less than the other variables, providing a brief justification would strengthen this decision.

Thank you for this valuable comment. We have revised the **Methodology** section to improve clarity by reducing repetitive descriptions related to data preparation and model training. In addition, we expanded the explanation for excluding **eigenvector centrality** from the final model. Specifically, we clarify that although six centrality measures were initially computed, eigenvector centrality consistently exhibited the weakest explanatory contribution and provided little additional discriminatory information beyond the other centrality measures. Excluding this variable reduced model complexity while preserving interpretability and ensuring a consistent predictor set for the comparative evaluation of Logistic Regression and the Graph Convolutional Network.

3. Results and Discussion

The results are presented clearly, and I appreciate that the authors evaluate both prediction performance and spatial consistency rather than relying on accuracy alone.

However, I believe the discussion could place greater emphasis on the practical implications of the findings.

For example:

How could planners use this model when making future land-use decisions?
Could this framework be applied to other cities with different street layouts?

Expanding this discussion would increase the practical value of the study.

Thank you for this valuable comment. We have expanded the **Discussion** section to emphasize the practical implications of the proposed framework. Specifically, we explain how the model can support urban planners in anticipating future residential-to-commercial transformation, informing zoning decisions, infrastructure investment, and sustainable corridor management. We also discuss the applicability of the proposed framework to other cities, highlighting that while the methodology is transferable to different urban contexts, local calibration using historical street-network and land-use data is recommended to account for differences in urban morphology, planning policies, and socio-economic conditions.

4. Figures

The figures generally support the discussion well.

However, some figures contain small labels that are difficult to read. Increasing the font size and improving the overall readability of these figures would make them easier to interpret.

Thank you for this valuable comment. We have carefully reviewed all figures and increased the font sizes of labels, legends, and annotations where necessary to improve readability. We also reviewed the overall figure formatting to ensure that all graphical elements are clear and easily interpretable in the revised manuscript.

5. References

The references are generally appropriate and current.

I would simply recommend performing one final review to ensure consistent formatting throughout the reference list, including capitalization and citation style.

Overall
Overall, this is an interesting study that addresses an important problem. The paper is well organized and easy to follow. With some improvements to the writing, a clearer explanation of the research gap, and a stronger discussion of the practical importance of the findings, the paper would be further strengthened and suitable for publication.

Thank you for this valuable comment. We have conducted a comprehensive review of the reference list and in-text citations to ensure consistency with the journal's formatting requirements, including capitalization, citation style, and overall reference formatting.

Reviewer #2: The manuscript is technically sound, and the presented data support its main conclusions.

The quantitative data are consistent with the authors' claims regarding model performance and feature importance.

Although only a single case study (Erbil) has been conducted using a binary classification (commercial vs. residential), the methodology is replicable provided the code is accessible. While the code's availability is declared as "Supporting Information", no specific repository link or DOI is explicitly provided within the current text of the manuscript.

Thank you for this valuable comment. We have revised the manuscript to explicitly include the Zenodo repository DOI for the supporting source code to facilitate reproducibility. The source code has been deposited in Zenodo (DOI: 10.5281/zenodo.21343914). As the manuscript is currently under review, the repository remains under embargo and will be made publicly accessible upon final acceptance of the manuscript, in accordance with the journal's data and code sharing policy.

**PLOS ONE: Edits requested on your submission PONE-D-26-24753R1 - [EMID:7497d7a19bd267d9]**

1. Thank you for updating your data availability statement. You note that your data are available within the Supporting Information files, but no such files have been included with your submission. At this time we ask that you please upload your minimal data set as a Supporting Information file, or to a public repository such as Figshare or Dryad.
   Please also ensure that when you upload your file you include separate captions for your supplementary files at the end of your manuscript.
   As soon as you confirm the location of the data underlying your findings, we will be able to proceed with the review of your submission.

We have uploaded the **minimal dataset** as a **Supporting Information** file. In addition, separate captions for all Supporting Information files have been included at the end of the manuscript, as requested.

2. Please upload a copy of Supporting Information Figures S1 Fig- S4 Fig which you refer to in your text on page 25.

We have uploaded all Supporting Information figures (S1 Fig–S4 Fig) as separate Supporting Information files. Corresponding captions for these figures are included at the end of the manuscript.

3. Thank you for your response. Could you also specify the source of the satellite or 3D imagery in the bottom-right quadrant of Figure 1?

The source of the satellite imagery shown in the bottom-right quadrant of Figure 1 has now been explicitly specified in the figure caption. The imagery was obtained from **Google Earth Pro,** while the map data used in the other panels are based on **OpenStreetMap data © OpenStreetMap contributors.**

Thank you for your time and consideration. We appreciate the opportunity to revise our manuscript and hope that these revisions satisfactorily address all of your comments.

**PLOS ONE: Edits requested on your submission PONE-D-26-24753R1 - [EMID:10c28071c6f5011b**

Thank you for the clarification regarding Google Earth imagery. We confirm that no Google Earth, Google Maps, or Google Street View imagery is reproduced in the revised manuscript or Supporting Information. All map figures currently included in the submission use OpenStreetMap data, and the figure captions have been revised to include the attribution recommended in the PLOS figure guidelines: “Contains information from OpenStreetMap and OpenStreetMap Foundation, which is made available under the Open Database License.” Google Earth Pro is mentioned in the Methods only because it was consulted as a historical visual reference during reconstruction of the 2004 street configuration; no Google imagery has been reproduced or incorporated into any submitted figure. We have revised the Methods to make this distinction explicit.
