# Supplementary material for: Graph convolutional network for modeling corridor-based urban transformation: Revealing spatial coherence in land-use change in Erbil
Source: PLoS One. 2026 Aug 27;21(8):e0357090. doi: 10.1371/journal.pone.0357090 (PMC13521350; doi:10.1371/journal.pone.0357090)
Supplement: Supplementary file 6 [file pone.0357090.s008.docx]

**Journal Requirements:**

If the reviewer comments include a recommendation to cite specific previously published works, please review and evaluate these publications to determine whether they are relevant and should be cited. There is no requirement to cite these works unless the editor has indicated otherwise. 
Please review your reference list to ensure that it is complete and correct. If you have cited papers that have been retracted, please include the rationale for doing so in the manuscript text, or remove these references and replace them with relevant current references.

Any changes to the reference list should be mentioned in the rebuttal letter that accompanies your revised manuscript. If you need to cite a retracted article, indicate the article’s retracted status in the References list and also include a citation and full reference for the retraction notice.

Response

Thank you for this guidance. We conducted a comprehensive review of all in-text citations and the complete reference list. Duplicate references were removed, mixed author–date citations were converted to numerical citations, incomplete and malformed bibliographic records were corrected, database-export text was removed, and the formatting and capitalization of all references were standardized. References that did not directly support the associated statements were removed or replaced with more relevant sources. All in-text citations and reference-list numbering were subsequently updated. The resulting changes are visible in the marked-up manuscript.

**Response to the Academic Editor**

**Manuscript ID: PONE-D-26-24753R1:**

Both reviewers recommend minor revision. Please revise the manuscript thoroughly and respond to all comments item by item.

This paper uses Logistic Regression and GCN to explore uneven commercial-residential development of street corridors, featuring clear structure and objective discussion. Required revisions are summarized as follows:

Expand the introduction, integrate literature review content and explicitly highlight the research gap.

Simplify repetitive descriptions in methodology, and supplement explanations for eliminating eigenvector centrality.

Enrich discussion on practical planning values and the model’s adaptability to different cities.

Increase font size of labels in all figures for better readability.

Standardize the citation format and capitalization of all references.

Provide the repository link or DOI of the supplementary research code for reproducibility.

After addressing all above points, the manuscript will be suitable for publication.

Dear Academic Editor,

We sincerely thank you and the reviewers for the constructive comments and positive assessment of our manuscript. We have revised the manuscript thoroughly and provide below a point-by-point response to each comment.

**Comment 1**

Expand the introduction, integrate literature review content and explicitly highlight the research gap.

**Response:**

Thank you for this valuable comment. We substantially expanded the Introduction and Literature Review to provide a stronger theoretical foundation for the study. The revised text now discusses the relationship between street-network structure, accessibility, commercial concentration, Logistic Regression, and graph-based urban modeling in greater depth.

We also added a dedicated subsection entitled **“2.4 Research gap,”** which explicitly identifies three gaps in the existing literature:

1. Limited historically grounded research on corridor-specific residential-to-commercial transformation.
2. Limited direct comparison between conventional statistical models and graph-based models using identical street-network predictors.
3. Insufficient consideration of the spatial coherence of predicted land-use transformation patterns.

The revised manuscript also explains clearly how the present study addresses these gaps through historical network reconstruction, direct comparison of Logistic Regression and GCN, and the use of spatial-coherence indicators alongside conventional classification metrics. These changes appear in Sections 1 and 2.1–2.4.

**Comment 2**

Simplify repetitive descriptions in methodology, and supplement explanations for eliminating eigenvector centrality.

**Response:**

Thank you. We revised the Methodology to remove repetitive descriptions and improve the organization and clarity of the analytical workflow. Repeated explanations concerning feature standardization, model validation, centrality calculation, and repeated train–test splitting were consolidated.

We also expanded the explanation for excluding eigenvector centrality. The revised manuscript now explains that feature screening was based on three complementary indicators: the bivariate correlation with the commercial label, the Logistic Regression coefficient, and the GCN feature-importance score. Eigenvector centrality showed the weakest contribution across all three indicators, including a correlation of −0.073, a Logistic Regression coefficient of −0.153, and a GCN importance score of 0.089. It was therefore excluded to reduce unnecessary model complexity while maintaining a common and informative predictor set for both models.

**Comment 3**

Enrich discussion on practical planning values and the model’s adaptability to different cities.

**Response:**

Thank you. The Discussion has been expanded to explain the practical planning value of the proposed framework as an early decision-support tool for corridor planning, zoning, infrastructure provision, traffic management, and the management of residential–commercial conflicts. We also clarified the conditions required for application in other cities, including local data preparation, parameter calibration, model retraining, and validation in relation to differences in urban morphology, planning regulation, transport systems, and socioeconomic conditions.

**Comment 4**

Increase font size of labels in all figures for better readability.

Thank you. The font sizes of all labels, legends, titles, annotations, scale information, and axis text in Figures 3–4 have been increased to improve readability. The revised figures have been checked at publication size to ensure that all textual elements remain clearly legible.

**Comment 5**

Standardize the citation format and capitalization of all references.

Thank you. We carefully reviewed all in-text citations and the complete reference list. Mixed author–date citations were converted to numerical citations, duplicate references were consolidated, bibliographic records were corrected, malformed DOI information and database-export text were removed, and capitalization and formatting were standardized consistently according to the journal’s reference style. References that did not directly support the associated statements were removed or replaced, and all citation numbers were updated accordingly.

We also screened the cited publications for retraction status and did not identify any retracted publications in the revised reference list. All reference-related changes are visible in the marked-up manuscript.

**Comment 6**

Provide the repository link or DOI of the supplementary research code for reproducibility.

**Response:**

Thank you. The repository DOI has been added to the revised manuscript. The research code and minimal dataset supporting the study are publicly available through Zenodo at:

[**https://doi.org/10.5281/zenodo.21343914**](https://doi.org/10.5281/zenodo.21343914)

The repository provides the materials required to support the reproducibility of the data preparation, model implementation, evaluation, and spatial-coherence analysis.

We sincerely appreciate the editor’s and reviewers’ constructive comments. The revisions have strengthened the theoretical framing, methodological clarity, practical relevance, readability, reference consistency, and reproducibility of the manuscript. We hope that the revised version now meets the publication requirements of PLOS ONE.
