# Supplementary material for: Graph convolutional network for modeling corridor-based urban transformation: Revealing spatial coherence in land-use change in Erbil
Source: PLoS One. 2026 Aug 27;21(8):e0357090. doi: 10.1371/journal.pone.0357090 (PMC13521350; doi:10.1371/journal.pone.0357090)
Supplement: Peer review history

# Peer review: Decision Letter 0

**DOI:** 10.1371/journal.pone.0357090.r001

**Authors:** Ming Sun

9 Jul 2026

Dear Dr. Amen,

Thank you for submitting your manuscript to PLOS ONE. After careful consideration, we feel that it has merit but does not fully meet PLOS ONE’s publication criteria as it currently stands. Therefore, we invite you to submit a revised version of the manuscript that addresses the points raised during the review process.

Please submit your revised manuscript by Aug 08 2026 11:59PM. If you will need more time than this to complete your revisions, please reply to this message or contact the journal office at plosone@plos.org. When you're ready to submit your revision, log on to https://www.editorialmanager.com/pone/ and select the 'Submissions Needing Revision' folder to locate your manuscript file.

- A letter that responds to each point raised by the academic editor and reviewer(s). You should upload this letter as a separate file labeled 'Response to Reviewers'.
- A marked-up copy of your manuscript that highlights changes made to the original version. You should upload this as a separate file labeled 'Revised Manuscript with Track Changes'.
- An unmarked version of your revised paper without tracked changes. You should upload this as a separate file labeled 'Manuscript'.

If you would like to make changes to your financial disclosure, please include your updated statement in your cover letter. Guidelines for resubmitting your figure files are available below the reviewer comments at the end of this letter.

If applicable, we recommend that you deposit your laboratory protocols in protocols.io to enhance the reproducibility of your results. Protocols.io assigns your protocol its own identifier (DOI) so that it can be cited independently in the future. For instructions see: https://journals.plos.org/plosone/s/submission-guidelines#loc-laboratory-protocols. Additionally, PLOS ONE offers an option for publishing peer-reviewed Lab Protocol articles, which describe protocols hosted on protocols.io. Read more information on sharing protocols at https://plos.org/protocols?utm_medium=editorial-email&utm_source=authorletters&utm_campaign=protocols.

As the corresponding author, your ORCID iD is verified in the submission system and will appear in the published article. PLOS supports the use of ORCID, and we encourage all coauthors to register for an ORCID iD and use it as well. Please encourage your coauthors to verify their ORCID iD within the submission system before final acceptance, as unverified ORCID iDs will not appear in the published article. *Only*  the individual author can complete the verification step; PLOS staff *cannot*  verify ORCID iDs on behalf of authors.

We look forward to receiving your revised manuscript.

Kind regards,

Ming Sun

Academic Editor

PLOS One

Journal Requirements:

When submitting your revision, we need you to address these additional requirements.

1. Please ensure that your manuscript meets PLOS ONE's style requirements, including those for file naming. The PLOS ONE style templates can be found at

https://journals.plos.org/plosone/s/file?id=wjVg/PLOSOne_formatting_sample_main_body.pdf and https://journals.plos.org/plosone/s/file?id=ba62/PLOSOne_formatting_sample_title_authors_affiliations.pdf

2. Please note that PLOS One has specific guidelines on code sharing for submissions in which author-generated code underpins the findings in the manuscript. In these cases, we expect all author-generated code to be made available without restrictions upon publication of the work. Please review our guidelines at https://journals.plos.org/plosone/s/materials-and-software-sharing#loc-sharing-code and ensure that your code is shared in a way that follows best practice and facilitates reproducibility and reuse.

3. Please provide a complete Data Availability Statement in the submission form, ensuring you include all necessary access information or a reason for why you are unable to make your data freely accessible. If your research concerns only data provided within your submission, please write "All data are in the manuscript and/or supporting information files" as your Data Availability Statement.

4. Please include a separate caption for each figure in your manuscript.

5. We note that Figure(s) 1, 3, 4 in your submission contain [map/satellite] images which may be copyrighted. All PLOS content is published under the Creative Commons Attribution License (CC BY 4.0), which means that the manuscript, images, and Supporting Information files will be freely available online, and any third party is permitted to access, download, copy, distribute, and use these materials in any way, even commercially, with proper attribution. For these reasons, we cannot publish previously copyrighted maps or satellite images created using proprietary data, such as Google software (Google Maps, Street View, and Earth). For more information, see our copyright guidelines: http://journals.plos.org/plosone/s/licenses-and-copyright.

We require you to either (1) present written permission from the copyright holder to publish these figures specifically under the CC BY 4.0 license, or (2) remove the figures from your submission:

a. You may seek permission from the original copyright holder of Figure(s) 1, 3, 4 to publish the content specifically under the CC BY 4.0 license.

We recommend that you contact the original copyright holder with the Content Permission Form (http://journals.plos.org/plosone/s/file?id=7c09/content-permission-form.pdf) and the following text:

“I request permission for the open-access journal PLOS ONE to publish XXX under the Creative Commons Attribution License (CCAL) CC BY 4.0 (http://creativecommons.org/licenses/by/4.0/). Please be aware that this license allows unrestricted use and distribution, even commercially, by third parties. Please reply and provide explicit written permission to publish XXX under a CC BY license and complete the attached form.”

Please upload the completed Content Permission Form or other proof of granted permissions as an "Other" file with your submission.

In the figure caption of the copyrighted figure, please include the following text: “Reprinted from [ref] under a CC BY license, with permission from [name of publisher], original copyright [original copyright year].”

b. If you are unable to obtain permission from the original copyright holder to publish these figures under the CC BY 4.0 license or if the copyright holder’s requirements are incompatible with the CC BY 4.0 license, please either i) remove the figure or ii) supply a replacement figure that complies with the CC BY 4.0 license. Please check copyright information on all replacement figures and update the figure caption with source information. If applicable, please specify in the figure caption text when a figure is similar but not identical to the original image and is therefore for illustrative purposes only.

The following resources for replacing copyrighted map figures may be helpful:

USGS National Map Viewer (public domain): http://viewer.nationalmap.gov/viewer/

The Gateway to Astronaut Photography of Earth (public domain): http://eol.jsc.nasa.gov/sseop/clickmap/

Maps at the CIA (public domain): https://www.cia.gov/library/publications/the-world-factbook/index.html and https://www.cia.gov/library/publications/cia-maps-publications/index.html

NASA Earth Observatory (public domain): http://earthobservatory.nasa.gov/

Landsat: http://landsat.visibleearth.nasa.gov/

USGS EROS (Earth Resources Observatory and Science (EROS) Center) (public domain): http://eros.usgs.gov/#

Natural Earth (public domain): http://www.naturalearthdata.com/

6. We note you have included a table to which you do not refer in the text of your manuscript. Please ensure that you refer to Table 1, 2, 3, 4 in your text; if accepted, production will need this reference to link the reader to the Table.

7. If the reviewer comments include a recommendation to cite specific previously published works, please review and evaluate these publications to determine whether they are relevant and should be cited. There is no requirement to cite these works unless the editor has indicated otherwise.

8. Please review your reference list to ensure that it is complete and correct. If you have cited papers that have been retracted, please include the rationale for doing so in the manuscript text, or remove these references and replace them with relevant current references. Any changes to the reference list should be mentioned in the rebuttal letter that accompanies your revised manuscript. If you need to cite a retracted article, indicate the article’s retracted status in the References list and also include a citation and full reference for the retraction notice.

Additional Editor Comments :

Thank you for submitting your manuscript on analyzing urban street commercial development using Graph Convolutional Networks (GCN). The review process indicates that your research topic addresses a critical issue in urban planning, and the attempt to apply GCN to capture spatial patterns is highly innovative. The reviewers highly praised the organization of the manuscript and its technical reliability, believing it has good potential for publication.

To further improve the quality of the manuscript and make it more suitable for the journal's publication standards, please refer to the following summary of revision suggestions:

1. Strengthen the Introduction and Literature Review:

Please substantially expand the introduction section. In the literature review, avoid merely listing previous studies; instead, focus on a comprehensive review that deeply analyzes the logical connections between studies. Please be sure to define the "research gap" more clearly, detailing the unresolved issues in existing research and the unique contributions of this study.

2. Optimize the Methodology Description:

The methodology section is generally clear, but it is recommended to streamline the repetitive descriptions regarding model training and data preparation. In addition, please provide further explanation for excluding "eigenvector centrality" from the final model. Even if its contribution is relatively small, providing clear statistical or theoretical justification will enhance the persuasiveness of the model.

3. Deepen the Results and Discussion:

Please expand the discussion on "practical implications" in the discussion section. For example, how can planners use this model to assist future land-use decisions? Is this framework applicable to other cities with drastically different street layouts? Addressing these points will significantly enhance the practical value of the research.

4. Figures, Tables, and Formatting Revisions:

Please check and increase the font size in the figures and tables to ensure that all labels are clear and readable. At the same time, please conduct a final comprehensive review of the reference format to ensure consistency in capitalization and citation style.

5. Data Availability:

Please clearly provide the repository link or DOI for the supporting code within the manuscript to ensure the reproducibility of the research.

We look forward to receiving your revised manuscript.

[Note: HTML markup is below. Please do not edit.]

Reviewers' comments:

Reviewer's Responses to Questions

**Comments to the Author**

1. Is the manuscript technically sound, and do the data support the conclusions?

Reviewer #1: Yes

Reviewer #2: Yes

**********

2. Has the statistical analysis been performed appropriately and rigorously?

Reviewer #1: Yes

Reviewer #2: Yes

**********

3. Have the authors made all data underlying the findings in their manuscript fully available?

The PLOS Data policy

Reviewer #1: Yes

Reviewer #2: No

**********

4. Is the manuscript presented in an intelligible fashion and written in standard English?

Reviewer #1: Yes

Reviewer #2: Yes

**********

Reviewer #1: Overview

The paper addresses a relevant urban planning problem by investigating why one particular street corridor experienced commercial development while nearby streets remained primarily residential. Comparing a traditional Logistic Regression model with a Graph Convolutional Network (GCN) is a great approach in my opinion, and the emphasis on capturing spatial patterns rather than focusing solely on prediction accuracy is a strength of the study.

The manuscript is well put together, and the methodology is explained in sufficient detail. The discussion is also balanced, as the authors acknowledge that the GCN does not outperform the Logistic Regression model in every performance metric.

However, there are some areas where the manuscript can be improved, and they are stated below.

Recommended revisions

1. Introduction and Research Gap

The introduction is a bit short and should be fleshed out further.

Also, the literature review reads more like a summary of previous studies than a discussion of how those studies relate to one another. I would suggest making this section more cohesive by synthesizing the literature rather than listing individual papers.

The research gap is mentioned, but it could be emphasized more clearly. I would recommend explaining in greater detail what existing studies have not addressed and how this work builds upon or differs from them.

2. Methodology

The methodology is generally well described and easy to understand.

That said, some implementation details are repeated throughout the section. For example, the discussion of the model training process and data preparation could be condensed without affecting the reader's understanding.

In addition, the decision to exclude eigenvector centrality from the final model could be explained in a little more detail. Although the paper mentions that it contributed less than the other variables, providing a brief justification would strengthen this decision.

3. Results and Discussion

The results are presented clearly, and I appreciate that the authors evaluate both prediction performance and spatial consistency rather than relying on accuracy alone.

However, I believe the discussion could place greater emphasis on the practical implications of the findings.

For example:

How could planners use this model when making future land-use decisions?

Could this framework be applied to other cities with different street layouts?

Expanding this discussion would increase the practical value of the study.

4. Figures

The figures generally support the discussion well.

However, some figures contain small labels that are difficult to read. Increasing the font size and improving the overall readability of these figures would make them easier to interpret.

5. References

The references are generally appropriate and current.

I would simply recommend performing one final review to ensure consistent formatting throughout the reference list, including capitalization and citation style.

Overall

Overall, this is an interesting study that addresses an important problem. The paper is well organized and easy to follow. With some improvements to the writing, a clearer explanation of the research gap, and a stronger discussion of the practical importance of the findings, the paper would be further strengthened and suitable for publication.

Reviewer #2: The manuscript is technically sound, and the presented data support its main conclusions.

The quantitative data are consistent with the authors' claims regarding model performance and feature importance.

Although only a single case study (Erbil) has been conducted using a binary classification (commercial vs. residential), the methodology is replicable provided the code is accessible. While the code's availability is declared as "Supporting Information", no specific repository link or DOI is explicitly provided within the current text of the manuscript.

**********

what does this mean?). If published, this will include your full peer review and any attached files.

If you choose “no”, your identity will remain anonymous but your review may still be made public.

**Do you want your identity to be public for this peer review?**  For information about this choice, including consent withdrawal, please see our Privacy Policy

Reviewer #1: No

Reviewer #2: No

**********

[NOTE: If reviewer comments were submitted as an attachment file, they will be attached to this email and accessible via the submission site. Please log into your account, locate the manuscript record, and check for the action link "View Attachments". If this link does not appear, there are no attachment files.]

To ensure your figures meet our technical requirements, please review our figure guidelines: https://journals.plos.org/plosone/s/figures

You may also use PLOS’s free figure tool, NAAS, to help you prepare publication quality figures: https://journals.plos.org/plosone/s/figures#loc-tools-for-figure-preparation.

NAAS will assess whether your figures meet our technical requirements by comparing each figure against our figure specifications.

# Author comment: Author response to Decision Letter 1

**DOI:** 10.1371/journal.pone.0357090.r002

24 Jul 2026

Journal Requirements:

1. Please ensure that your manuscript meets PLOS ONE's style requirements, including those for file naming. The PLOS ONE style templates can be found at

https://journals.plos.org/plosone/s/file?id=wjVg/PLOSOne_formatting_sample_main_body.pdf and https://journals.plos.org/plosone/s/file?id=ba62/PLOSOne_formatting_sample_title_authors_affiliations.pdf

Thank you for bringing this to our attention. We have carefully reviewed the manuscript and revised it to comply with the PLOS ONE formatting and style requirements, including the required file naming conventions. The revised files have been prepared according to the journal's formatting guidelines.

2. Please note that PLOS One has specific guidelines on code sharing for submissions in which author-generated code underpins the findings in the manuscript. In these cases, we expect all author-generated code to be made available without restrictions upon publication of the work. Please review our guidelines at https://journals.plos.org/plosone/s/materials-and-software-sharing#loc-sharing-code and ensure that your code is shared in a way that follows best practice and facilitates reproducibility and reuse.

Thank you for this requirement. We have reviewed the PLOS ONE code-sharing policy. The Python code developed for data preprocessing, graph construction, model implementation, and evaluation has already been uploaded to Zenodo, and a DOI has been reserved (DOI: 10.5281/zenodo.21343914). The repository will remain private during the peer-review process and will be made publicly available after the manuscript receives final acceptance, in accordance with the journal's code-sharing policy.

3. Please provide a complete Data Availability Statement in the submission form, ensuring you include all necessary access information or a reason for why you are unable to make your data freely accessible. If your research concerns only data provided within your submission, please write "All data are in the manuscript and/or supporting information files" as your Data Availability Statement.

The processed dataset and the source code supporting the findings of this study have been deposited in Zenodo (DOI: 10.5281/zenodo.21343914). The repository is currently private during peer review and will be made publicly available upon final acceptance of the manuscript, in accordance with the journal's data and code sharing policy.

4. Please include a separate caption for each figure in your manuscript.

Thank you for this comment. We have carefully reviewed all figures and ensured that each figure has a separate, complete, and descriptive caption in accordance with the PLOS ONE formatting guidelines. The figure captions have been revised where necessary to improve clarity and comply with the journal's requirements

5. We note that Figure(s) 1, 3, 4 in your submission contain [map/satellite] images which may be copyrighted. All PLOS content is published under the Creative Commons Attribution License (CC BY 4.0), which means that the manuscript, images, and Supporting Information files will be freely available online, and any third party is permitted to access, download, copy, distribute, and use these materials in any way, even commercially, with proper attribution. For these reasons, we cannot publish previously copyrighted maps or satellite images created using proprietary data, such as Google software (Google Maps, Street View, and Earth). For more information, see our copyright guidelines: http://journals.plos.org/plosone/s/licenses-and-copyright.

Thank you for bringing this issue to our attention. We have carefully reviewed the figures. Figure 1 has been revised to remove the Google Earth imagery and ensure compliance with the PLOS ONE copyright and licensing requirements. Figures 3 and 4 were originally created in QGIS using OpenStreetMap data and do not contain any Google Earth or Google Maps imagery. Therefore, no changes to these figures were necessary. Appropriate attribution to OpenStreetMap has been included in the figure captions.

6. We note you have included a table to which you do not refer in the text of your manuscript. Please ensure that you refer to Table 1, 2, 3, 4 in your text; if accepted, production will need this reference to link the reader to the Table.

Thank you for this comment. We have reviewed the tables and revised their formatting to comply with the PLOS ONE guidelines. All tables are presented as editable tables with separate, descriptive titles and have been placed in the manuscript immediately after their first citation in the text, in accordance with the journal's formatting requirements.

7. If the reviewer comments include a recommendation to cite specific previously published works, please review and evaluate these publications to determine whether they are relevant and should be cited. There is no requirement to cite these works unless the editor has indicated otherwise.

Thank you for this comment. We carefully reviewed all references suggested by the reviewers and evaluated their relevance to the present study. Relevant references have been incorporated into the revised manuscript where appropriate.

8. Please review your reference list to ensure that it is complete and correct. If you have cited papers that have been retracted, please include the rationale for doing so in the manuscript text, or remove these references and replace them with relevant current references. Any changes to the reference list should be mentioned in the rebuttal letter that accompanies your revised manuscript. If you need to cite a retracted article, indicate the article’s retracted status in the References list and also include a citation and full reference for the retraction notice

Thank you for this comment. We have carefully reviewed the reference list to ensure that it is complete, accurate, and up to date. The references have been checked for correctness, and any necessary revisions have been made. We also confirmed that none of the cited references have been retracted.

Additional Editor Comments :

1. Strengthen the Introduction and Literature Review:

Please substantially expand the introduction section. In the literature review, avoid merely listing previous studies; instead, focus on a comprehensive review that deeply analyzes the logical connections between studies. Please be sure to define the "research gap" more clearly, detailing the unresolved issues in existing research and the unique contributions of this study.

Thank you for this valuable comment. We have substantially revised both the Introduction and the Literature Review. The Introduction has been expanded to provide a stronger background, clearly state the study objectives, and highlight the novelty and contributions of the research. The Literature Review has been reorganized into thematic sections that critically synthesize previous studies rather than listing them individually. In addition, we added a dedicated Research Gap section that clearly identifies the unresolved issues in the literature and explains how the present study addresses these gaps through the comparison of Logistic Regression and Graph Convolutional Networks and the incorporation of spatial coherence as an additional evaluation criterion.

2. Optimize the Methodology Description:

The methodology section is generally clear, but it is recommended to streamline the repetitive descriptions regarding model training and data preparation. In addition, please provide further explanation for excluding "eigenvector centrality" from the final model. Even if its contribution is relatively small, providing clear statistical or theoretical justification will enhance the persuasiveness of the model.

Thank you for this valuable comment. We have revised the Methodology section to improve clarity and reduce repetitive descriptions related to data preparation and model training. In addition, we have expanded the explanation for excluding eigenvector centrality, providing both theoretical and empirical justification. Specifically, eigenvector centrality exhibited weak discriminative power in the study corridor and showed limited contribution to model performance, making its inclusion unnecessary while improving model interpretability.

3. Deepen the Results and Discussion:

Please expand the discussion on "practical implications" in the discussion section. For example, how can planners use this model to assist future land-use decisions? Is this framework applicable to other cities with drastically different street layouts? Addressing these points will significantly enhance the practical value of the research.

Thank you for this valuable comment. We have expanded the Discussion section by adding a dedicated discussion on the practical implications of the proposed framework. The revised manuscript now explains how the model can support urban planners in anticipating corridor-based commercial transformation, informing zoning and infrastructure decisions, and guiding sustainable land-use management. We also discuss the transferability of the proposed framework to other cities, highlighting its general applicability while acknowledging the need for local calibration to account for differences in urban morphology, planning policies, and socio-economic conditions.

4. Figures, Tables, and Formatting Revisions:

Please check and increase the font size in the figures and tables to ensure that all labels are clear and readable. At the same time, please conduct a final comprehensive review of the reference format to ensure consistency in capitalization and citation style.

Thank you for this valuable comment. We have reviewed all figures and tables and increased the font sizes where necessary to improve the readability of labels, legends, and annotations. In addition, we conducted a comprehensive review of the reference list and in-text citations to ensure consistency with the journal's formatting requirements, including capitalization, citation style, and reference formatting throughout the manuscript.

5. Data Availability:

Please clearly provide the repository link or DOI for the supporting code within the manuscript to ensure the reproducibility of the research.

Thank you for this comment. We have revised the manuscript to include the Zenodo repository DOI for the supporting code to facilitate reproducibility. The source code has been deposited in Zenodo (DOI: 10.5281/zenodo.21343914). The repository is currently under embargo and will be made publicly accessible upon final acceptance of the manuscript.

Review Comments to the Author

Reviewer #1:

1. Introduction and Research Gap

The introduction is a bit short and should be fleshed out further.

Also, the literature review reads more like a summary of previous studies than a discussion of how those studies relate to one another. I would suggest making this section more cohesive by synthesizing the literature rather than listing individual papers.

The research gap is mentioned, but it could be emphasized more clearly. I would recommend explaining in greater detail what existing studies have not addressed and how this work builds upon or differs from them.

Thank you for this valuable comment. We have substantially revised both the Introduction and the Literature Review. The Introduction has been expanded to provide a stronger background, clearly define the study objectives, and emphasize the novelty and contributions of the research. The Literature Review has been reorganized into thematic sections that critically synthesize previous studies rather than simply summarizing individual works. In addition, a dedicated Research Gap section has been added to clearly identify the unresolved issues in the existing literature and explain how the present study addresses these gaps through historical street-network reconstruction, the comparison of Logistic Regression and Graph Convolutional Networks, and the incorporation of spatial coherence as an additional model evaluation criterion.

2. Methodology

The methodology is generally well described and easy to understand.

That said, some implementation details are repeated throughout the section. For example, the discussion of the model training process and data preparation could be condensed without affecting the reader's understanding.

In addition, the decision to exclude eigenvector centrality from the final model could be explained in a little more detail. Although the paper mentions that it contributed less than the other variables, providing a brief justification would strengthen this decision.

Thank you for this valuable comment. We have revised the Methodology section to improve clarity by reducing repetitive descriptions related to data preparation and model training. In addition, we expanded the explanation for excluding eigenvector centrality from the final model. Specifically, we clarify that although six centrality measures were initially computed, eigenvector centrality consistently exhibited the weakest explanatory contribution and provided little additional discriminatory information beyond the other centrality measures. Excluding this variable reduced model complexity while preserving interpretability and ensuring a consistent predictor set for the comparative evaluation of Logistic Regression and the Graph Convolutional Network.

3. Results and Discussion

The results are presented clearly, and I appreciate that the authors evaluate both prediction performance and spatial consistency rather than relying on accuracy alone.

However, I believe the discussion could place greater emphasis on the practical implications of the findings.

For example:

How could planners use this model when making future land-use decisions?

Could this framework be applied to other cities with different street layouts?

Expanding this discussion would increase the practical value of the study.

Thank you for this valuable comment. We have expanded the Discussion section to emphasize the practical implications of the proposed framework. Specifically, we explain how the model can support urban planners in anticipating future residential-to-commercial transformation, informing zoning decisions, infrastructure investment, and sustainable corridor management. We also discuss the applicability of the proposed framework to other cities, highlighting that while the methodology is transferable to different urban contexts, local calibration using historical street-network and land-use data is recommended to account for differences in urban morphology, planning policies, and socio-economic conditions.

4. Figures

The figures generally support the discussion well.

However, some figures contain small labels that are difficult to read. Increasing the font size and improving the overall readability of these figures would make them easier to interpret.

Thank you for this valuable comment. We have carefully reviewed all figures and increased the font sizes of labels, legends, and annotations where necessary to improve readability. We also reviewed the overall figure formatting to ensure that all graphical elements are clear and easily interpretable in the revised manuscript.

5. References

The references are generally appropriate and current.

I would simply recommend performing one final review to ensure consistent formatting throughout the reference list, including capitalization and citation style.

Overall

Overall, this is an interesting study that addresses an important problem. The paper is well organized and easy to follow. With some improvements to the writing, a clearer explanation of the research gap, and a stronger discussion of the practical importance of the findings, the paper would be further strengthened and suitable for publication.

Thank you for this valuable comment. We have conducted a comprehensive review of the reference list and in-text citations to ensure consistency with the journal's formatting requirements, including capitalization, citation style, and overall reference formatting.

Reviewer #2: The manuscript is technically sound, and the presented data support its main conclusions.

The quantitative data are consistent with the

Attachment Submitted filename: Response to Reviewers.docx

# Editor report: Decision Letter 1

**DOI:** 10.1371/journal.pone.0357090.r003

**Authors:** Ming Sun

29 Jul 2026

Dear Dr. Amen,

Thank you for submitting your manuscript to PLOS ONE. After careful consideration, we feel that it has merit but does not fully meet PLOS ONE’s publication criteria as it currently stands. Therefore, we invite you to submit a revised version of the manuscript that addresses the points raised during the review process.

Please submit your revised manuscript by Aug 28 2026 11:59PM. If you will need more time than this to complete your revisions, please reply to this message or contact the journal office at plosone@plos.org. When you're ready to submit your revision, log on to https://www.editorialmanager.com/pone/ and select the 'Submissions Needing Revision' folder to locate your manuscript file.

- A letter that responds to each point raised by the academic editor and reviewer(s). You should upload this letter as a separate file labeled 'Response to Reviewers'.
- A marked-up copy of your manuscript that highlights changes made to the original version. You should upload this as a separate file labeled 'Revised Manuscript with Track Changes'.
- An unmarked version of your revised paper without tracked changes. You should upload this as a separate file labeled 'Manuscript'.

If you would like to make changes to your financial disclosure, please include your updated statement in your cover letter. Guidelines for resubmitting your figure files are available below the reviewer comments at the end of this letter.

If applicable, we recommend that you deposit your laboratory protocols in protocols.io to enhance the reproducibility of your results. Protocols.io assigns your protocol its own identifier (DOI) so that it can be cited independently in the future. For instructions see: https://journals.plos.org/plosone/s/submission-guidelines#loc-laboratory-protocols. Additionally, PLOS ONE offers an option for publishing peer-reviewed Lab Protocol articles, which describe protocols hosted on protocols.io. Read more information on sharing protocols at https://plos.org/protocols?utm_medium=editorial-email&utm_source=authorletters&utm_campaign=protocols.

As the corresponding author, your ORCID iD is verified in the submission system and will appear in the published article. PLOS supports the use of ORCID, and we encourage all coauthors to register for an ORCID iD and use it as well. Please encourage your coauthors to verify their ORCID iD within the submission system before final acceptance, as unverified ORCID iDs will not appear in the published article. *Only*  the individual author can complete the verification step; PLOS staff *cannot*  verify ORCID iDs on behalf of authors.

We look forward to receiving your revised manuscript.

Kind regards,

Ming Sun

Academic Editor

PLOS One

Journal Requirements:

If the reviewer comments include a recommendation to cite specific previously published works, please review and evaluate these publications to determine whether they are relevant and should be cited. There is no requirement to cite these works unless the editor has indicated otherwise.

Please review your reference list to ensure that it is complete and correct. If you have cited papers that have been retracted, please include the rationale for doing so in the manuscript text, or remove these references and replace them with relevant current references. Any changes to the reference list should be mentioned in the rebuttal letter that accompanies your revised manuscript. If you need to cite a retracted article, indicate the article’s retracted status in the References list and also include a citation and full reference for the retraction notice.

Additional Editor Comments:

Both reviewers recommend minor revision. Please revise the manuscript thoroughly and respond to all comments item by item.

This paper uses Logistic Regression and GCN to explore uneven commercial-residential development of street corridors, featuring clear structure and objective discussion. Required revisions are summarized as follows:

Expand the introduction, integrate literature review content and explicitly highlight the research gap.

Simplify repetitive descriptions in methodology, and supplement explanations for eliminating eigenvector centrality.

Enrich discussion on practical planning values and the model’s adaptability to different cities.

Increase font size of labels in all figures for better readability.

Standardize the citation format and capitalization of all references.

Provide the repository link or DOI of the supplementary research code for reproducibility.

After addressing all above points, the manuscript will be suitable for publication.

[NOTE: If reviewer comments were submitted as an attachment file, they will be attached to this email and accessible via the submission site. Please log into your account, locate the manuscript record, and check for the action link "View Attachments". If this link does not appear, there are no attachment files.]

To ensure your figures meet our technical requirements, please review our figure guidelines: https://journals.plos.org/plosone/s/figures

You may also use PLOS’s free figure tool, NAAS, to help you prepare publication quality figures: https://journals.plos.org/plosone/s/figures#loc-tools-for-figure-preparation.

NAAS will assess whether your figures meet our technical requirements by comparing each figure against our figure specifications.

# Author comment: Author response to Decision Letter 2

**DOI:** 10.1371/journal.pone.0357090.r004

31 Jul 2026

Journal Requirements:

If the reviewer comments include a recommendation to cite specific previously published works, please review and evaluate these publications to determine whether they are relevant and should be cited. There is no requirement to cite these works unless the editor has indicated otherwise.

Please review your reference list to ensure that it is complete and correct. If you have cited papers that have been retracted, please include the rationale for doing so in the manuscript text, or remove these references and replace them with relevant current references.

Any changes to the reference list should be mentioned in the rebuttal letter that accompanies your revised manuscript. If you need to cite a retracted article, indicate the article’s retracted status in the References list and also include a citation and full reference for the retraction notice.

Response

Thank you for this guidance. We conducted a comprehensive review of all in-text citations and the complete reference list. Duplicate references were removed, mixed author–date citations were converted to numerical citations, incomplete and malformed bibliographic records were corrected, database-export text was removed, and the formatting and capitalization of all references were standardized. References that did not directly support the associated statements were removed or replaced with more relevant sources. All in-text citations and reference-list numbering were subsequently updated. The resulting changes are visible in the marked-up manuscript.

Response to the Academic Editor

Manuscript ID: PONE-D-26-24753R1:

Both reviewers recommend minor revision. Please revise the manuscript thoroughly and respond to all comments item by item.

This paper uses Logistic Regression and GCN to explore uneven commercial-residential development of street corridors, featuring clear structure and objective discussion. Required revisions are summarized as follows:

Expand the introduction, integrate literature review content and explicitly highlight the research gap.

Simplify repetitive descriptions in methodology, and supplement explanations for eliminating eigenvector centrality.

Enrich discussion on practical planning values and the model’s adaptability to different cities.

Increase font size of labels in all figures for better readability.

Standardize the citation format and capitalization of all references.

Provide the repository link or DOI of the supplementary research code for reproducibility.

After addressing all above points, the manuscript will be suitable for publication.

Dear Academic Editor,

We sincerely thank you and the reviewers for the constructive comments and positive assessment of our manuscript. We have revised the manuscript thoroughly and provide below a point-by-point response to each comment.

Comment 1

Expand the introduction, integrate literature review content and explicitly highlight the research gap.

Response:

Thank you for this valuable comment. We substantially expanded the Introduction and Literature Review to provide a stronger theoretical foundation for the study. The revised text now discusses the relationship between street-network structure, accessibility, commercial concentration, Logistic Regression, and graph-based urban modeling in greater depth.

We also added a dedicated subsection entitled “2.4 Research gap,” which explicitly identifies three gaps in the existing literature:

1. Limited historically grounded research on corridor-specific residential-to-commercial transformation.

2. Limited direct comparison between conventional statistical models and graph-based models using identical street-network predictors.

3. Insufficient consideration of the spatial coherence of predicted land-use transformation patterns.

The revised manuscript also explains clearly how the present study addresses these gaps through historical network reconstruction, direct comparison of Logistic Regression and GCN, and the use of spatial-coherence indicators alongside conventional classification metrics. These changes appear in Sections 1 and 2.1–2.4.

Comment 2

Simplify repetitive descriptions in methodology, and supplement explanations for eliminating eigenvector centrality.

Response:

Thank you. We revised the Methodology to remove repetitive descriptions and improve the organization and clarity of the analytical workflow. Repeated explanations concerning feature standardization, model validation, centrality calculation, and repeated train–test splitting were consolidated.

We also expanded the explanation for excluding eigenvector centrality. The revised manuscript now explains that feature screening was based on three complementary indicators: the bivariate correlation with the commercial label, the Logistic Regression coefficient, and the GCN feature-importance score. Eigenvector centrality showed the weakest contribution across all three indicators, including a correlation of −0.073, a Logistic Regression coefficient of −0.153, and a GCN importance score of 0.089. It was therefore excluded to reduce unnecessary model complexity while maintaining a common and informative predictor set for both models.

Comment 3

Enrich discussion on practical planning values and the model’s adaptability to different cities.

Response:

Thank you. The Discussion has been expanded to explain the practical planning value of the proposed framework as an early decision-support tool for corridor planning, zoning, infrastructure provision, traffic management, and the management of residential–commercial conflicts. We also clarified the conditions required for application in other cities, including local data preparation, parameter calibration, model retraining, and validation in relation to differences in urban morphology, planning regulation, transport systems, and socioeconomic conditions.

Comment 4

Increase font size of labels in all figures for better readability.

Thank you. The font sizes of all labels, legends, titles, annotations, scale information, and axis text in Figures 3–4 have been increased to improve readability. The revised figures have been checked at publication size to ensure that all textual elements remain clearly legible.

Comment 5

Standardize the citation format and capitalization of all references.

Thank you. We carefully reviewed all in-text citations and the complete reference list. Mixed author–date citations were converted to numerical citations, duplicate references were consolidated, bibliographic records were corrected, malformed DOI information and database-export text were removed, and capitalization and formatting were standardized consistently according to the journal’s reference style. References that did not directly support the associated statements were removed or replaced, and all citation numbers were updated accordingly.

We also screened the cited publications for retraction status and did not identify any retracted publications in the revised reference list. All reference-related changes are visible in the marked-up manuscript.

Comment 6

Provide the repository link or DOI of the supplementary research code for reproducibility.

Response:

Thank you. The repository DOI has been added to the revised manuscript. The research code and minimal dataset supporting the study are publicly available through Zenodo at:

https://doi.org/10.5281/zenodo.21343914

The repository provides the materials required to support the reproducibility of the data preparation, model implementation, evaluation, and spatial-coherence analysis.

We sincerely appreciate the editor’s and reviewers’ constructive comments. The revisions have strengthened the theoretical framing, methodological clarity, practical relevance, readability, reference consistency, and reproducibility of the manuscript. We hope that the revised version now meets the publication requirements of PLOS ONE.

Attachment Submitted filename: Response_to_Reviewers_auresp_2.docx

# Editor report: Decision Letter 2

**DOI:** 10.1371/journal.pone.0357090.r005

**Authors:** Ming Sun

12 Aug 2026

Graph Convolutional Network for Modeling Corridor-Based Urban Transformation: Revealing Spatial Coherence in Land-Use Change in Erbil

PONE-D-26-24753R2

Dear Dr. ,

We’re pleased to inform you that your manuscript has been judged scientifically suitable for publication and will be formally accepted for publication once it meets all outstanding technical requirements.

Within one week, you’ll receive an e-mail detailing the required amendments. When these have been addressed, you’ll receive a formal acceptance letter and your manuscript will be scheduled for publication.

An invoice will be generated when your article is formally accepted. Please note, if your institution has a publishing partnership with PLOS and your article meets the relevant criteria, all or part of your publication costs will be covered. Please make sure your user information is up-to-date by logging into Editorial Manager at Editorial Manager® and clicking the ‘Update My Information' link at the top of the page. For questions related to billing, please contact billing support.

If your institution or institutions have a press office, please notify them about your upcoming paper to help maximize its impact. If they’ll be preparing press materials, please inform our press team as soon as possible -- no later than 48 hours after receiving the formal acceptance. Your manuscript will remain under strict press embargo until 2 pm Eastern Time on the date of publication. For more information, please contact onepress@plos.org.

Kind regards,

Ming Sun

Academic Editor

PLOS One

Additional Editor Comments (optional):

Verify the formatting of figures, tables, and open-access repository links, as well as reference lists.

Reviewers' comments:

# Editor report: Acceptance letter

**DOI:** 10.1371/journal.pone.0357090.r006

**Authors:** Ming Sun

PONE-D-26-24753R2

PLOS One

Dear Dr. Amen,

I'm pleased to inform you that your manuscript has been deemed suitable for publication in PLOS One. Congratulations! Your manuscript is now being handed over to our production team.

At this stage, our production department will prepare your paper for publication. This includes ensuring the following:

* All references, tables, and figures are properly cited

* All relevant supporting information is included in the manuscript submission,

* There are no issues that prevent the paper from being properly typeset

You will receive further instructions from the production team, including instructions on how to review your proof when it is ready. Please keep in mind that we are working through a large volume of accepted articles, so please give us a few days to review your paper and let you know the next and final steps.

Lastly, if your institution or institutions have a press office, please let them know about your upcoming paper now to help maximize its impact. If they'll be preparing press materials, please inform our press team within the next 48 hours. Your manuscript will remain under strict press embargo until 2 pm Eastern Time on the date of publication. For more information, please contact onepress@plos.org.

You will receive an invoice from PLOS for your publication fee after your manuscript has reached the completed accept phase. If you receive an email requesting payment before acceptance or for any other service, this may be a phishing scheme. Learn how to identify phishing emails and protect your accounts at https://explore.plos.org/phishing.

If we can help with anything else, please email us at customercare@plos.org.

Thank you for submitting your work to PLOS One and supporting open access.

Kind regards,

PLOS ONE Editorial Office Staff

on behalf of

Dr. Ming Sun

Academic Editor

PLOS One
